# The rise of resilient healthcare research during COVID-19: scoping review of empirical research

**DOI:** 10.1186/s12913-023-09839-0

**Published:** 2023-08-07

**Authors:** Louise A Ellis, Maree Saba, Janet C Long, Hilda Bø Lyng, Cecilie Haraldseid-Driftland, Kate Churruca, Siri Wiig, Elizabeth Austin, Robyn Clay-Williams, Ann Carrigan, Jeffrey Braithwaite

**Affiliations:** 1 Centre for Healthcare Resilience and Implementation Science, Australian Institute of Health Innovation, Macquarie University, Sydney, Australia; 2 Centre Faculty of Health Sciences, SHARE - Centre for Resilience in Healthcare, University of Stavanger, Stavanger, Norway

**Keywords:** Healthcare research, Health systems, Resilience, Resilience in healthcare, Resilient healthcare, Safety-II

## Abstract

**Background:**

The COVID-19 pandemic has presented many multi-faceted challenges to the maintenance of service quality and safety, highlighting the need for resilient and responsive healthcare systems more than ever before. This review examined empirical investigations of Resilient Health Care (RHC) in response to the COVID-19 pandemic with the aim to: identify key areas of research; synthesise findings on capacities that develop RHC across system levels (micro, meso, macro); and identify reported adverse consequences of the effort of maintaining system performance on system agents (healthcare workers, patients).

**Methods:**

Three academic databases were searched (Medline, EMBASE, Scopus) from 1st January 2020 to 30th August 2022 using keywords pertaining to: systems resilience and related concepts; healthcare and healthcare settings; and COVID-19. Capacities that developed and enhanced systems resilience were synthesised using a hybrid inductive-deductive thematic analysis.

**Results:**

Fifty publications were included in this review. Consistent with previous research, studies from high-income countries and the use of qualitative methods within the context of hospitals, dominated the included studies. However, promising developments have been made, with an emergence of studies conducted at the macro-system level, including the development of quantitative tools and indicator-based modelling approaches, and the increased involvement of low- and middle-income countries in research (LMIC). Concordant with previous research, eight key resilience capacities were identified that can support, develop or enhance resilient performance, namely: structure, alignment, coordination, learning, involvement, risk awareness, leadership, and communication. The need for healthcare workers to constantly learn and make adaptations, however, had potentially adverse physical and emotional consequences for healthcare workers, in addition to adverse effects on routine patient care.

**Conclusions:**

This review identified an upsurge in new empirical studies on health system resilience associated with COVID-19. The pandemic provided a unique opportunity to examine RHC in practice, and uncovered emerging new evidence on RHC theory and system factors that contribute to resilient performance at micro, meso and macro levels. These findings will enable leaders and other stakeholders to strengthen health system resilience when responding to future challenges and unexpected events.

**Supplementary Information:**

The online version contains supplementary material available at 10.1186/s12913-023-09839-0.

## Background

Resilient Health Care (RHC) is defined as the ability of a system to adjust its functioning prior to, during, or following changes and disturbances, so that it can sustain required operations under both expected and unexpected conditions [1]. The COVID-19 pandemic presented challenges that healthcare systems must address to maintain service quality and safety, highlighting the need for resilient and responsive healthcare systems more than ever before [2]. Healthcare practitioners, managers, and policy makers had to suddenly, and dramatically, adapt in order to absorb the shock of the pandemic and coordinate the capacities needed to deal with its impact. Since the onset of the pandemic, ‘health systems resilience’ has emerged as a key concept in global public health with the World Health Organization (WHO) publishing several papers [3–7] on the importance of building and strengthening health emergency preparedness and responsiveness to future epidemics and shocks.

The application of resilience thinking to healthcare is however not new, with RHC being first proposed by Eric Hollnagel in 2011 [8] to describe the application of resilience engineering [9] and disaster resilience [10, 11] to healthcare. RHC acknowledges the complex adaptive nature of healthcare, recognising the adaptive and transformative capabilities that enable healthcare systems to continue to perform their functions in the face of challenges [12, 13]. Despite its conceptual appeal, there have been challenges in translating the principles of RHC into concrete improvements, with compelling examples remaining scarce [14].

The importance of RHC is reflected in the growing number of reviews on the topic [13, 15, 16]. Although these reviews identified that the RHC literature has been predominantly conceptual, rather than empirical [13, 15, 16], empirical applications of RHC have increased. A systematic review conducted prior to the pandemic identified 71 empirical studies on health system resilience from 2008 to 2019, with 62% of these published in the last two years of the review (i.e., from 2017 to 2019) [15]. However, much of this existing empirical literature has focused on clinical microsystems at the ‘sharp end’ and how frontline healthcare professionals within hospital settings collectively adapt, ‘work around’, or enable things to go well [2, 13], with a lack of empirical studies particularly at the meso and macro-levels (i.e., government, national, international) [14]. Qualitative research methods have also predominated in the empirical studies [13, 15], reflecting that priorities have been placed on gaining in-depth understanding of everyday clinical work at the micro-level.

Another noteworthy gap in the RHC literature is the limited discussions on how ‘individual agents’ (e.g., doctors, nurses) [17] within the health system may be personally affected by their efforts to maintain system resilience [18]. However, the time appears ripe for this issue to be explored in the context of RHC, particularly in light of the COVID-19 pandemic, which has caused major disruptions across all system levels and created a need for ongoing adaptation by healthcare workers, which many suggest has resulted in widespread mental health issues and burnout amongst these workers [19, 20].

### The present study

**Fig. 1:**
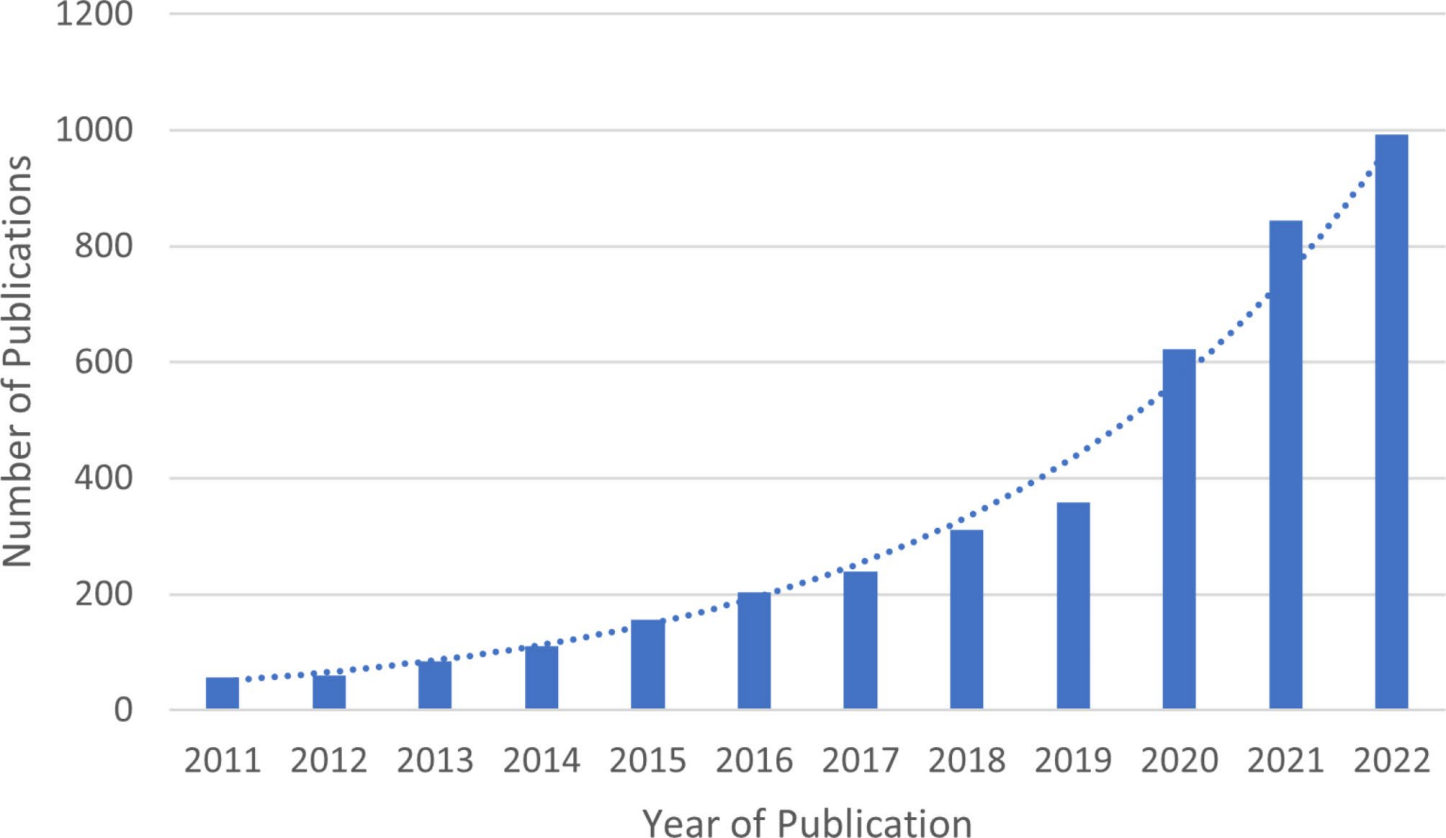
Increased publications in PubMed using the search term “health systems resilience” in titles or abstracts

Interest in RHC has accelerated since the onset of the COVID-19 pandemic, as indicated by the sharp increase in the number of publications in ‘health systems resilience’ since 2020 (Fig. 1**).** With the growth in empirical contributions in this field, it is timely to examine the published empirical research to determine the status of the field and identify whether there is any further evidence on how to generate or strengthen resilient performance to manage future pandemics and emergencies. Understanding factors that develop or enhance RHC is critical to developing strategies and tools for strengthening their resilience [12]. For this review, we defined an empirical study as one that reports primary or secondary data gathered by means of a specific methodological approach [21]. The objective of this study was to conduct a scoping review of empirical investigations of RHC in response to the COVID-19 pandemic with four key aims:

1. Map out the empirical research within the resilient healthcare domain across all system levels (micro, meso, macro).
2. Identify the key areas of research, including study designs and research methods that have been employed.
3. Synthesise findings on factors (capacities, actions, or strategies) that developed or enhanced resilient performance.
4. Identify any reported findings on consequences of maintaining system performance on system agents (healthcare workers, patients).

## Methods

The review followed a pre-determined protocol, developed in accordance with the Preferred Reporting Items of Systematic Review and Meta-Analyses Extension for Scoping Reviews (PRISMA-ScR) [22, 23] (also see PRISMA-ScR in Supplementary File 1). A scoping review method was used; a method which is used to examine the extent, range and nature of work on this topic and to identify gaps and provide suggestions to improve future directions for RHC research [24]. Quality assessments were not undertaken, as the aim was to examine the full breadth of the empirical literature, consistent with general aims and methodology of scoping reviews [25].

### Search strategy

Three academic databases (Medline, EMBASE, Scopus) were searched from 1st January 2020 to 30th August 2022. The search strategy consisted of terms pertaining to: systems resilience (e.g., resilient healthcare) and related concepts (e.g., Safety-II); healthcare (e.g., health care) and healthcare settings (e.g., primary care, hospital); and COVID-19. The search strategy was adapted for each database as necessary (see Supplementary File 2 for the complete search strategy, using Ovid MEDLINE as an example). The search strategy was developed in consultation with an academic research librarian and was reviewed by all authors prior to execution.

### Inclusion and exclusion criteria

Articles were included if they were: (a) published between the onset of COVID-19 (from 1st January 2020) and 30 August 2022, (b) in the English language, (c) peer-reviewed publications, (d) had an explicit focus on healthcare or health systems resilience in the context of COVID-19, and (e) were empirical studies. Studies that only mentioned “resilience” briefly, were concerned with individual or psychological resilience (e.g., the psychological wellbeing of healthcare workers) rather than systems-resilience or were not conducted in the context of COVID-19 were excluded. Study protocols, review papers, journal commentaries, and editorials were also excluded, as were studies not in English.

### Eligibility screening

Reference details (including abstracts) were downloaded into the reference management software Endnote X9 and then exported to Rayyan QCRI for title and abstract screening. Seven reviewers (LAE, MS, JCL, KC, EA, LT, DT) screened the title/abstracts to determine their inclusion against the criteria, with 5% of the retrieved publications being independently screened by the entire review team to ensure consistent inclusion. Any discrepancies among reviewers’ judgements were reviewed by two authors (LAE and MS) with JB available for consultation if and as needed.

### Data extraction

Data from included studies meeting inclusion criteria were extracted into a custom workbook in Microsoft Excel. Full-text screening was conducted initially by two independent reviewers (LT, DT), with LAE and MS subsequently duplicating the full-text review process, with any discrepancies being discussed and resolved in consultation with JB. The extraction workbook included data items on: [1] publication details (paper title, year, output type); [2] study context (e.g., hospital, primary care); [3] system level (micro: healthcare practitioner; meso: management, organisation; and/or macro: government, national, international); [4] study design (quantitative, qualitative, mixed methods); [5] study data type (primary or secondary); [6] data collection method/s (quantitative, qualitative, mixed-methods); [7] conceptual framework, model, or theory used; [8] resilience measure or tool used; [9] factors (capacities, actions, or strategies) that developed and enhanced systems resilience; and [10] reported negative consequences of resilient performance on system agents (healthcare workers, patients).

### Data synthesis and analysis

A data-based convergent synthesis was employed [26]; where quantitative data were transformed into categories or themes, and summarised through narrative techniques [27]. Country of the corresponding author was coded by income classification based on World Bank definitions of gross national income per capita. The three categories were low (< US$1085), middle (US$1086–13,205), and high income (> US$13,205) [28]. Data collection methods were categorised as qualitative, quantitative or mixed methods, with specific data collection methods (e.g., interviews, surveys) also extracted and examined.

The factors that supported, developed or enhanced systems resilience were initially identified through an inductive thematic approach [29] by two authors (LAE, MS). Themes and sub-themes were then discussed and agreed by the researchers using an iterative process. Upon further analysis and reflection of the themes, it was clear that a number of the themes aligned with the ‘capacities’ for resilience outlined by Lyng et al. [30]. Therefore, in the next phase, a deductive approach was taken where the themes and sub-themes were mapped to eight of the resilience ‘capacities’. Some minor amendments were made based upon differences in themes identified in the literature included in the present review compared with the capacities. Two of the ‘capacities’ outlined by Lyng et al. [30], namely ‘competence’ and ‘facilitators’, were not included owing to the lack of data mapping to these themes, as identified from the initial inductive analysis. Themes and subthemes were cross-referenced across all studies to ensure that the revised thematic map captured the meaning across all the included studies. The last phase involved defining the themes (see Table 1 for definitions as applied in this study). Consequences of maintaining resilient performance were similarly identified using an inductive thematic approach [29] by two authors (LAE, MS).

**Table 1:** Definition of factors that developed and enhanced systems resilience

Resilience Capacity	Definition
Structure	The structures that support work and practice within the organisation, including resources, equipment, technology, and governance systems.
Learning	The provision of learning activities and learning opportunities.
Alignment	The various adaptations introduced to bring in line the different external and situational circumstances of what is required at a given time.
Coordination	How teams facilitate and organise work within and between teams and organisations.
Leadership	How leaders facilitate, support, motivate and contribute to the organisation.
Risk awareness	The extent to which an organisation understands and is prepared for risk.
Involvement	The involvement of patients and families in decision-making and adaptations to meet the needs of patients.
Communication	The systems used to translate information within and between teams and organisations.

Adapted from Lyng et al. [30]

## Results

### Overview of included studies

**Fig. 2:**
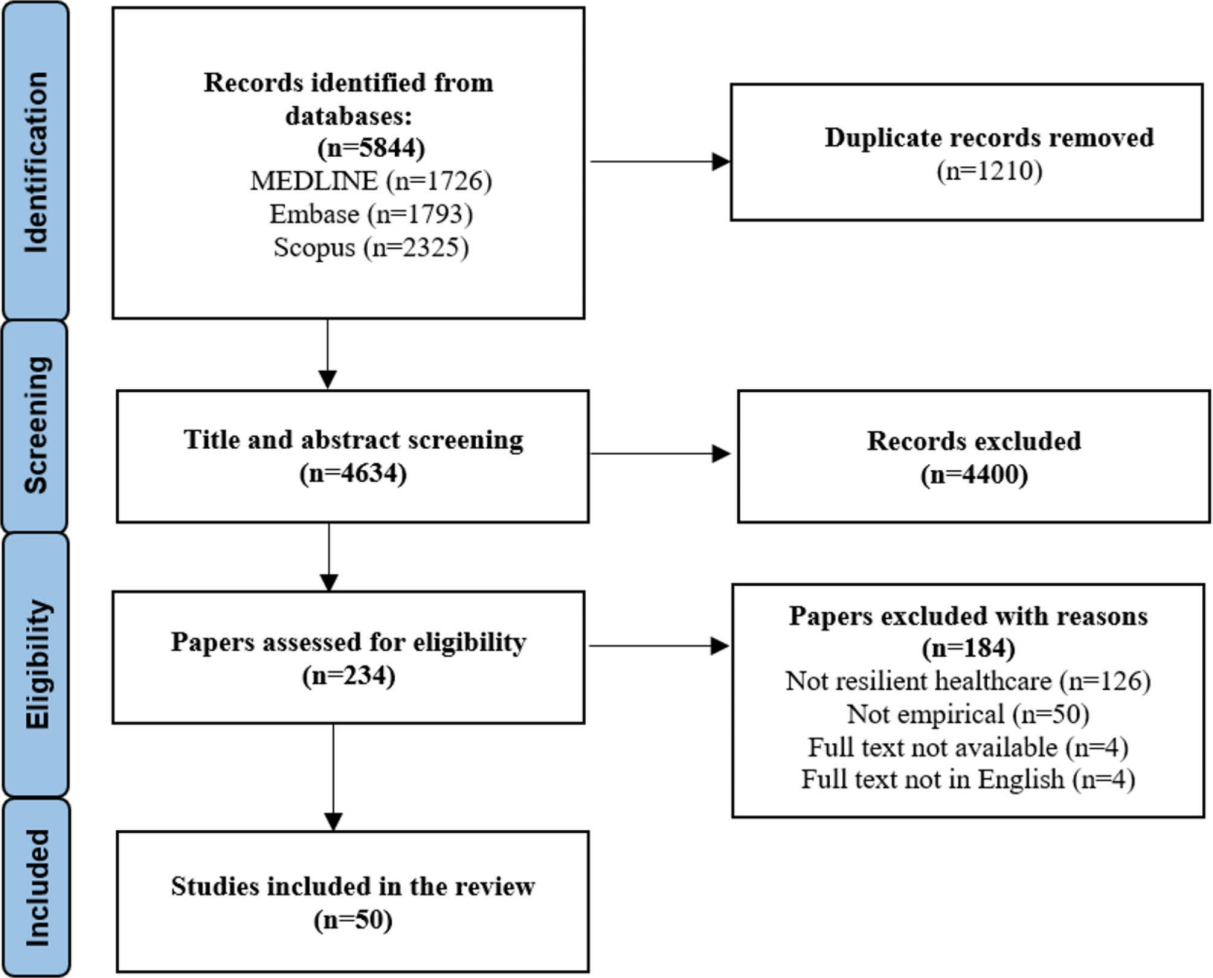
PRISMA flow diagram for study selection process

The initial search retrieved a total of 5844 publications. After removing duplicates, 4634 remained for title and/or abstract review. Following title and/or abstract screening, 4404 publications were discarded as they did not meet the inclusion criteria. Based on the full-text assessment, a further 184 publications did not meet the inclusion criteria, resulting in 50 publications included in this review (see Supplementary File 3 for included articles). Figure 2 demonstrates the inclusion and exclusion of papers at each stage of the screening process.

### Summary characteristics of the included studies

A summary of the key characteristics of the included papers is provided in Table 2. The 50 studies were spread widely, across 45 different journals, with Safety Science (n = 3, 6.0%) and the International Journal of Health Policy and Management (n = 3, 6.0%) being the most popular. The source location was also spread widely, across 25 different countries, with most corresponding authors from the United Kingdom (n = 8, 16.0%), followed by the United States (n = 6, 12.0%). Although most studies were restricted to high-income countries (n = 34, 68%), a notable number of corresponding authors were identified from low- and middle-income countries (LMIC) (n = 16, 32.0%), and with four (8%) of these being from Brazil.

Close to half (n = 20, 40%) of the studies were conducted in the context of hospitals, which generally involved hospital healthcare workers and/or hospital leaders as participants. Four studies (8%) [31–34] were specifically focused on supply chain issues related to medical supply availability in the context of system adaptability and resilience, and its impact on the healthcare system more broadly. Of the studies conducted in the context of community and specialised care (n = 15, 30%), a number were focused on the resilient performance of aged care services [35–37] or community mental health services [38–40]. Primary care was a setting in seven studies (14%), with a focus on the perspectives of primary care providers in relation to healthcare system resilience [38, 41–46]. Over half of the studies were classified as being at the meso level (n = 29, 58%) of the healthcare system, with fewer studies being at the micro level (n = 17, 34%) or macro level (n = 18, 36%). Notably, eleven (61%) of these macro-level studies, incorporated data from multiple countries, such as a comparison study of health system resilience across six European countries, a comparison study of government actions and their relation to systems resilience between Canada and Australia, and an indicator-based analysis of risk and resilience that incorporated ‘big data’ from 11 countries.

Three-quarters of the studies were qualitative (n = 39, 78%), seven were mixed-methods (14%) and four were quantitative (8%). Although most studies utilised primary data alone (n = 39, 78%), seven studies relied on secondary datasets (14%), such as existing big data sources [47] and questionnaire data [48, 49], and a smaller number used both primary and secondary datasets (n = 4, 8%).

**Table 2:** Summary of key characteristics of included publications

Classification	Number of papers	%
**Country of corresponding author**		
United Kingdom	8	16
United States	6	12
Brazil	4	8
Canada	4	8
Germany	3	6
Other	25	50
**Country income classification**		
High-income	34	68
Middle-income	13	26
Low-income	3	6
Study context*		
Hospital	20	40
Community and specialised care	15	30
Government and policy	8	16
Primary care (general practice)	7	14
Supply chain management	4	8
System level*		
Micro	17	34
Meso	29	58
Macro	18	36
**Study methods**		
Qualitative methods	39	78
Quantitative methods	4	8
Mixed-methods	7	14
**Study data type**		
Primary data	39	78
Secondary data	7	14
Both primary and secondary data	4	8

*Columns do not sum to n = 50 or 100% due to overlap in some categories

### Data collection methods and tools to assess RHC

Most of the studies collected data from direct sources (i.e., where participants directly express their experience of how work takes place in practice) [16], and included interviews (n = 32, 64%), surveys (n = 15, 30%) or focus groups (n = 3, 6%). A smaller number of studies included indirect sources, such as document analysis (n = 9, 18%), observations (n = 4, 8%), and/or simulation (n = 2, 4%). One-third of studies developed and/or used tools to study RHC (n = 17, 34%); of these, over half employed researcher-developed questionnaires to assess or understand resilient performance (n = 11, 65%), three adopted a ‘big data’ indicator-based approach to assess systems resilience for emergency preparedness, two studies drew on the more commonly regarded Functional Resonance Analysis Method (FRAM) [50], and one study used observation tools based on the “Mayo high performance team scale” [51] and the “Scrub Practitioners List of Intra-operative Non-Technical Skills (SPLINTS)” [52].

Over half the researcher-developed questionnaires (n = 7, 64%) were based on a conceptual framework, including Hollnagel’s [53] ‘four cornerstones of resilience’ [54], Anderson et al.’s [55] Integrated Resilience Attributes Framework [56], Bueno et al.’s [57] guidelines for coping with complexity [58], Macrae and Wiig’s [59] resilience framework [35], the WHO’s [60] fundamental ‘building blocks’ of health systems [61, 62] and the WHO’s hospital readiness checklist [63, 64]. Three additional survey studies lacking a conceptual framework collected predominantly open-ended questionnaire data on how everyday clinical work is being performed during the pandemic (i.e., work-as-done), via the perceptions and experiences of healthcare workers [32, 43], using inductive content analysis, and to confirm or corroborate any emerging themes identified from interview data [65]. One final questionnaire tool was developed to assess hospital inventory management, including the impact of COVID-19 on the availability of supply and the processes established to enhance supply chain resilience [31].

### Capacities that developed and enhanced resilient performance

**Fig. 3:**
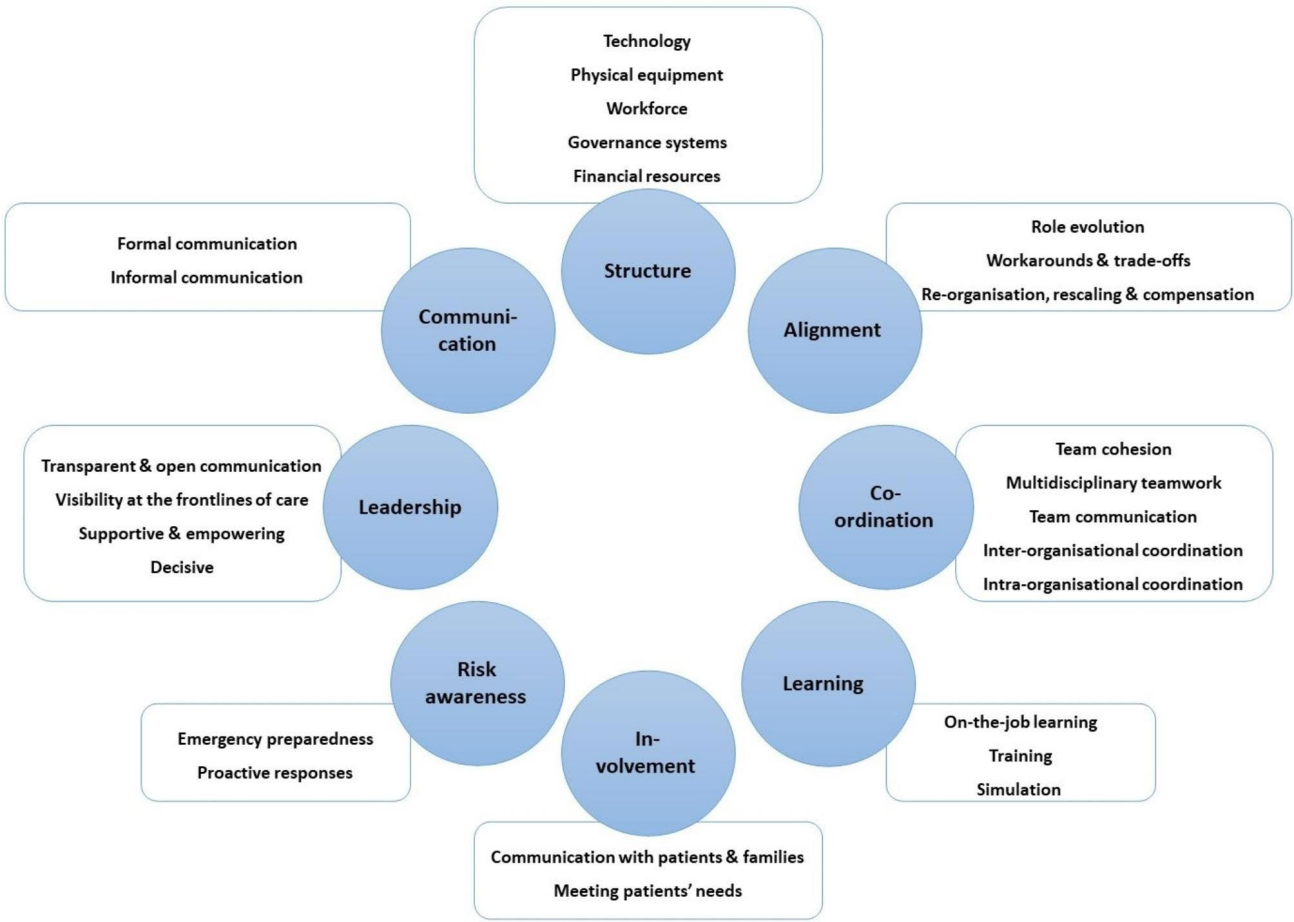
Resilience capacities and related sub-themes

Based on the analysis of the included studies, eight key factors or capacities were identified at different system levels to develop or enhance resilient performance, as outlined in the following section. In this section, the eight resilience capacities have been discussed sequentially from the capacity that occurred most prevalently within the included studies to the capacity that occurred least prevalently, namely: structure, alignment, coordination, learning, involvement, risk awareness, leadership, and communication. Figure 3 provides a visual summary of the eight factors and their sub-themes (also see Supplementary File 4 giving examples for each subtheme).

#### Structure

Structure as a capacity for resilience was identified in more than four-fifths of included studies (n = 37, 74%) and referred to the structures that support work and practice within healthcare organisations. Across the included studies in this review, five sub-themes contributed to structural capacity, including: technology, physical equipment, workforce, governance systems and financial resources.

The most prevalent among the subthemes, *technology* (n = 27, 54%), concerned how software and hardware were utilised during the pandemic to support the continued delivery of regular healthcare services, as well as COVID-specific responses. Several studies highlighted a spike in the use of different technologies to enable the provision of patient care in different settings [41, 44, 66, 67]. For example, Gifford et al. [66] reported the way in which wards and outpatient clinics rapidly converted to “digital” wards involving e-health, video and phone consultations. Alternatively, in one study from Canada [68], a lack of appropriate technology impeded resilient performance, with the rapid but “piecemeal” adoption of multiple virtual care technologies during COVID-19 resulting in systems that duplicated administrative work for healthcare professionals.

Access to *physical equipment* (n = 18, 36%), such as personal protective equipment (PPE), or flexible workspaces, was another prevalent subtheme across the studies. In many instances it was the lack of availability of this equipment, particularly during the early stages of the pandemic, that impeded the COVID response [36, 46, 69]. However, several studies reported the way in which organisations rapidly responded by adapting equipment levels, including how and where they sourced physical equipment, as well as their novel repurposing of in-house equipment [35] and wards to create additional capacity [66].

*Workforce* (n = 11, 22%) involved access to staff, workforce stability, and the designation of roles and responsibilities. Some of these studies highlighted challenges in recruitment, and how understaffing affected resilient performance [39, 69], as there was both increased demand for healthcare and staff shortages due to workers contracting COVID-19. Organisational adaptations to promote resilience and address this issue included the reassignment of staff to other parts of the hospital [56] and expanding their reach in hiring new staff, which included the provision of financial incentives [39] and the re-employment of recently retired staff [66].

*Governance systems and protocols* (n = 19, 38%) involved the development of new policies, or modification of existing ones, to support the many changes in work practices during the pandemic. In some instances, these policies were devised at a macro-level [39], while in others they were more locally developed [70]. Along with this, *financial resources* (n = 5, 10%), involved funding changes wrought by the pandemic, including the allocation of funding to support COVID care delivery [71], as well as the financial implications of the pandemic in lost revenue due to a reduction in consultations, particularly identified for small healthcare providers [41].

#### Alignment

Alignment as a capacity for resilient performance referred to the adaptation of practices in response to the ever-changing problems posed by the COVID-19 pandemic [30]. Identified in over half of the included studies (n = 30, 60%), the alignment capacity included three subthemes: role evolution; micro-level workarounds and trade-offs; and meso- to macro-level re-structuring, rescaling and compensation strategies.

*Role evolution* (n = 13, 26%) concerned how roles and responsibilities of healthcare workers and leaders changed or expanded in response to the ongoing challenges of the pandemic. Healthcare managers and leaders were asked to step into different functions; for example, in crisis management, communications and crisis responses [66]. Clinical staff also needed to expand their responsibilities, extend their working hours, and were redeployed to other wards to fulfill staff shortages and meet patient demands [66]. A smaller number of staff were redeployed to special COVID-19 teams, providing direct care to infected patients [56, 66, 72] and healthcare leaders worked from home [56], to limit further staff exposure to the virus. The change in workspace and role, as well as the pressing needs of COVID-19 infected patients, meant that staff had to be trained in new procedures and practices; for instance, redeployed physiotherapists into intensive care units and research staff into clinical roles [71]. Although redeployment sometimes caused stress and uncertainty, with the additional challenge of unfamiliar workspaces and colleagues, redeployment was also perceived as an opportunity for positive career development and empowerment [65].

The COVID-19 pandemic introduced a need for healthcare workers to improvise and develop solutions to unexpected and frequent problems, introducing *workarounds* and *trade-offs* (n = 19, 38%) at the micro-system level. Several studies highlighted how healthcare workers developed unique and creative workarounds at the front-line to help them cope with ongoing challenges [35, 41, 66, 70]. For example, workarounds intended to ease the impact of the pandemic on patients and their families included: decorating PPE masks, using dance as a greeting instead of hugging, and providing outdoor concerts for patients [35, 70]. Additionally, some studies described staff changes in prioritization, also known in the RHC literature as trade-offs, directing their capacity to where it was needed most. This meant that scheduled surgeries and regular care were scaled down to increase capacity such as in intensive care units (ICUs) and emergency departments [66]. The risk of infection also introduced trade-offs for community health workers, as home visits were no longer allowed; instead, community health workers began to take on administrative tasks at health clinics [43].

The COVID-19 pandemic also led to alignment strategies at the meso- and macro-levels, as COVID-19 provided exceptional demands for all parts of the health system. *Re-organisation*, *rescaling* and *compensation* (n = 19, 38%) strategies at the organizational level included arranging for COVID-19 treatment areas, wards, assessment clinics, COVID-19 teams, and new types of administration [71]. Furthermore, new emergency plans, policies, and safety standards, such as providing separate entrances and exits at nursing homes [35], were initiated to limit spread of the virus [69]. Unlike their traditional way of working, strategies for restructuring, rescaling, and compensation often had to be created “on the go” due to the unpredictability and unfamiliarity of the situation [39]. However, two studies highlighted [58, 66] that healthcare systems can cope more effectively with future crises by factoring in “slack resources” at an organizational level and collective level (i.e., network or national), thereby ensuring the continued availability of critical medical supplies, equipment, and human resources. Likewise, supply chain resilience studies described the adoption of “buffering” and “bridging” strategies [34], along with “strategic purchasing” [33], to ensure continued healthcare supply and equipment availability across the healthcare system.

#### Coordination

Coordination as a capacity for resilience referred to how teams facilitated and organised work within and between teams and organisations. Identified in over half (n = 28, 56%) of studies in this review, coordination included the following five subthemes: team cohesion; multidisciplinary teamwork; team communication; inter-organisational coordination; and intra-organisational coordination. In terms of *team cohesion* (n = 10, 20%), building a supportive and cohesive team was regarded as an important factor in developing and sustaining resilient performance, particularly at the clinical micro-systems of care. Several studies expressed increased “connection” [72], “collaboration” [39, 70–72] and a “sense of camaraderie” [70] among teams during the pandemic as they “rallied together” [40] and “worked together toward a common goal” [70]. Traditional clinical hierarchies were also reported as less important during delivery of care [72], leading to enhanced team dynamics and coordination [73]. Three studies also highlighted the role of “peer support” [56, 65, 69] as co-workers provided reassurance and supported staff wellbeing.

*Multidisciplinary teamwork* (n = 10, 20%) was also emphasised as critical in developing and sustaining resilient performance during the pandemic. Multidisciplinary teamwork was often initially made more difficult (e.g., in cases where teams were physically divided, or fewer staff on site), however, healthcare workers adapted [70] and used creative solutions to make multidisciplinary care more accessible [44, 56, 70, 74]. Hodgins et al. [71] described the “breaking down of silos”, with staff from different disciplines “coming together” to support each other and sustain resilience. Ensuring that *team communication* (n = 5, 10%) remained open within and between teams was also critical to ensure teams remained connected and up to date with the ever-changing situation, as well as helping to facilitate the support process [39, 42, 72, 75].

Along with evolving processes and workflows, *inter-organisational coordination* (n = 15, 30%) and teamwork evolved throughout the pandemic. Several studies outlined the establishment of multidisciplinary teams being formed at the hospital throughout various stages of the pandemic (e.g., COVID-19-management teams, emergency response teams, specialist care teams) [40, 63, 66, 72, 74] to enable rapid response and care to changing situations. Resilient performance was fostered by experienced teams and inter-organisational collaborations who adapted and worked together, with tenacity and creativity, in ways that previously had not been required [36, 67, 70]. *Intra-organisational coordination* (n = 7, 14%) was also described as critical during the pandemic, providing a buffer to combat resource shortages (e.g., workforce, equipment, knowledge). Services were reported as drawing on both new and pre-existing relationships to overcome barriers to care [34, 36, 74].

#### Learning

Learning as a capacity for resilient performance described the facilitation of knowledge acquisition, through the provision of learning activities and opportunities [30]. Learning was identified in just under half of the included studies (n = 21, 42%), and consisted of three subthemes: on-the-job learning, training, and simulation.

*On-the-job learning* (n = 9, 18%) became particularly important during the COVID-19 pandemic. Exposure to new situations, equipment, and regulations, forced healthcare personnel to continuously adjust and learn during everyday work; for example, the appropriate use of protective equipment [35] or the prompt need to develop decision-making and communication skills [69]. The novelty of the situation, with lack of standardized treatment plans often brought a trial-and-error approach whereby healthcare personnel became prepared through on-going daily training sessions [72], and through shared knowledge and experience [65, 69, 72].

*Training***(**n = 15, 30%) referred to more planned and scheduled efforts to increase knowledge and preparedness through organised learning efforts, such as courses, simulations, e-learning, and workshops [56]. These training efforts had different aims than those before the pandemic, ranging from technical skill development, such as medical equipment [69], to non-technical skills such as management skills [66, 70]. The training sessions often took place at in-house-learning arenas such as simulation centres or labs, but also online learning resources were applied to reach a boarder audience and avoid spread of the virus [70].

*Simulation* (n = 3, 6%) as a novel training approach was identified in a small number of studies to increase preparedness to the COVID- 19 situation. Simulations allowed for interdisciplinary teams to train together and become confident in their technical and non-technical skills [75]. New simulation teams were created, and schedules developed to run consecutive training sessions, allowing for a large part of the healthcare personnel to be involved in the training [71].

#### Involvement

Involvement, as a key capacity for resilience in healthcare, referred to how the organisation involved and supported effective interactions between different system actors such as family, patients, and other stakeholders [35]. Meaningful involvement was evident in over one-third (n = 18, 36%) of the included studies and identified through two subthemes: communication with patients and families, and meeting patients’ needs.

Technology and roles were leveraged as a means for *communication with patients and families* (n = 14, 28%) and ensured patients and families continued to be engaged with care delivery during the COVID-19 pandemic. Changes to protocols and policy intending to reduce the transmission of COVID-19 (e.g., physical distancing, reduced capacity) required healthcare personnel to adjust how patients and families were meaningfully involved in care from primarily face-to-face to remote platforms. For example, teleconsultation technology was used to facilitate patient access to care services including a 24-hour helpline [76], and new systems to provide care services with the means to monitor and support patients remotely [41]. Technology was also used during the ‘no visitor policy’ to allow COVID-19 patients to connect with their family and medical staff when in isolation [66]. Volunteer networks and patient navigators were also used to extend services and connect healthcare providers with families [70, 77], with posters and flyers on public noticeboards also used to share important health related information with families with limited literacy [70].

Practices and processes were adapted to ensure the health system was *meeting patients’ needs* (n = 10, 20%) during the pandemic. Changes to practices and processes were intended to mitigate unintended consequences of reduced or remote interaction service delivery methods to manage COVID-19 (e.g., postponing care, contagion fear) and ensure care delivery strategies had the capacity to address the needs of patients and that patient access to care was maintained [38]. For example, nursing specific care delivery processes were adapted to overcome difficulties in involving patients and family members to meet the immediate needs of patients [72] and practices were reorganised to comply with hygienic guidelines, thus enabling patients with acute non-COVID-19 needs to access care [41].

#### Risk awareness

Risk awareness as a capacity for resilient performance, enhances a system’s resilience when understanding and responding to potential adverse events [30]. Identified in over one-third of included studies (n = 18, 36%), risk awareness comprised two subthemes: emergency preparedness; and proactive responses.

From the early stages of the pandemic, *emergency preparedness* (n = 10, 20%) to COVID-19 was fundamental in planning and arranging strategies to meet the constant demands on the health system [72]. The development and continued “fine-tuning” of emergency preparedness plans [39, 41, 42, 61, 78] has been described as both important and necessary [39]. Emergency plans were attuned to strengthen other resilience capacities, such as streamlining communication systems [42, 78], governance structures (78) and decision-making structures, to ensure the “continued, effective operation of the health system” [42]. One study also highlighted that the knowledge and experience gained from COVID-19 has led to ongoing conversations at a leadership level around emergency preparedness for any future crises [39].

Monitoring and proactive response (n = 16, 32%) referred to the understanding of situational risks to allow for proactive responses at all healthcare levels [30]. Early responses to the pandemic were often described as “ad-hoc”, but as the pandemic progressed, indicators and responses were monitored internationally [36, 72, 79] to assess risk, enabling proactive rather than reactive responses to problems [36, 72, 79]. Several studies outlined the implementation of an emergency taskforce [36, 61, 72] which met daily to evaluate emerging evidence [36], or devised new prevention strategies [61] or digital healthcare supply chain strategy [78]. Other studies discussed organisational infrastructure to prepare for the future risk of an outbreak, such as tracking COVID-19 positive individuals within hospitals, monitoring PPE levels [71] and developing plans for housing patients at alternative locations [39].

#### Leadership

Leadership (n = 16, 32%) as a resilient capacity demonstrated the important contribution of leaders to both their employees and the broader healthcare organisation. Four subthemes were identified that contributed to the leadership capacity: transparent and open communication; visibility at the frontlines of care; supportive and empowering; and decisive leadership.

*Transparent and open communication* (n = 4, 8%) from leaders was noted as crucial in dealing with the pandemic. Leaders were required to distribute a continuous flow of information from national and regional authorities to the front-line staff through various channels [35], providing updates as new information became known. In general, frontline staff found this information to be both useful and supportive [72].

Increased *visibility* of leaders at the frontlines of care (n = 8, 16%) was also identified as important. For example, Lyng et al. [35] reported that leaders at Norwegian nursing homes heavily affected by the pandemic altered their daily work schedules so they could be present at the frontlines of care. On the other hand, where staff expressed an absence of effective and visible leadership, there was a sense of “mistrust in leaders”, generating a negative environment [65].

Resilient performance was also associated with leaders who were s*upportive and empowering* (n = 8, 16%). Along with visibility at the frontlines, leaders were reported as providing logistical support, expressing “appreciation of hard work”, offering “motivations and rewards” to continue, and “empowerment” to adapt to the changed conditions [69]. At one large healthcare organisation, leaders were reported as showing genuine concern for their staff’s mental and physical wellbeing [39], and at others, as providing reassurance to “frightened and exhausted” staff [36].

The value of *decisive leadership* (n = 10, 20%) in enabling resilient performance during the pandemic was reported in several studies. The ongoing changing nature of the pandemic required leaders to make rapid decisions [36], be flexible yet decisive [39], take proactive steps, and adopt a more hierarchical “military” style of command [80]. For example, with the constant stream of new updates and information comings to leaders, they needed to adopt a “learning mindset” to respond effectively and be willing to change course if warranted by the new information [66].

#### Communication

In almost one-third of included studies (n = 15, 30%), communication was identified as a key capacity for resilient performance and included the systems of communication used to translate information within and between teams and organisations. Two main systems of communication were identified: formal communication, such as information communication technology [72] and policies sent via email [70]; and informal communication, such as social media apps [56, 65, 70].

Several studies reported the utilisation of *formal communication* systems (n = 10, 20%) during the COVID-19 pandemic. It was widely accepted that the pandemic necessitated the rapid upskilling and education of staff and patients, and it was crucial that information was accurately resourced and disseminated [71]. For example, rapidly changing information from national and regional authorities was circulated, and healthcare executives provided daily COVID-19 updates via several communication platforms, such as the staff intranet and emails [35, 70, 71, 80]. Providers also received regular policy and procedural updates (e.g., infection control) as more information from regulatory bodies became available [72]. However, some communication gaps were also identified; for example, a lack of communication aligned with rapidly changing protocols that increased the difficulty of remaining informed [56]. Challenges included a lack of intra-and inter professional communication between other units [56], a lack of access to technology and inconsistent information [81].

*Informal communication* (n = 10, 20%) was also reported among many of the included studies, commonly involving the development of group chats via social media apps, such as WhatsApp. These communication tools facilitated the sharing of information, such as policy and procedural change, and helped to provide emotional support and load sharing at the start of the pandemic among teams [35, 56, 65, 70, 76].

### Consequences on system agents

It was clear from the included studies that navigating the challenges of the COVID-19 pandemic, which came with the need to constantly learn and make adaptations in response to unexpected variation and changes, came at a personal cost to healthcare workers, particularly to those at the frontlines of care. Nine (18%) of the included studies reported that the increased workload and strenuous work conditions had negative *physical consequences* on healthcare workers [54, 56, 61, 67–69, 79, 81, 82]. For example, nurses reported increased “tiredness”, “exhaustion”, “muscle weakness” and “loss of appetite”, during the pandemic as a result of working longer shifts, often without breaks, while being “weighed down by PPE equipment” [67, 69].

The pandemic also exposed staff to stressful situations, which had considerable *emotional consequences* on staff, a theme identified in one-third of studies (n = 17, 34%). During the early stages of the pandemic, COVID-19 created an environment of uncertainty and fear among the population as a whole, but especially among front line workers [43], who expressed fear of dying from COVID-19, depression, worry, and frustration, among other psychological complaints [69]. Leaders were no different, with one study reporting that COVID-19 had also been emotionally demanding for staff in administrative and clinical leadership roles, with “constant exposure to vicarious trauma seeping into their personal and family time outside of work” [39]. Facing simultaneous pressures of physical and emotional demands, resulted in increased incidence of severe stress, emotional exhaustion, and burnout amongst healthcare workers [69]. One study further identified the cyclical nature of the problem, with burnt out healthcare workers on stress-leave causing greater staff shortages and increased workload for those remaining at work [56].

Several studies also identified that despite the healthcare system demonstrating several capacities to exhibit resilient performance in response to COVID-19, negative “spillover effects” were exhibited on routine patient care [44]. For example, Lotta et al. noted that the physical distancing requirements and mandatory use of PPE undermined everyday clinical work, with healthcare workers not being able to maintain contact with families [43]. Additionally, Akinyemi et al. [80] detailed that the COVID-19 pandemic negatively impacted service delivery in the healthcare system, for example, through disruptions to the appointment system and emergency and routine care services, which affected patient access to healthcare.

## Discussion

RHC broadly refers as a system’s capacity to maintain or restore its functions despite disruptions caused by external factors [59]. RHC does not focus on an individual’s coping and resilience capacity but rather on the factors and tools that enable the workers, teams, department and organisation to adapt and cope effectively in different situations [16]. RHC is a theoretically attractive concept, with its positive focus on how ‘things go right’ rather than wrong, and as evidenced by the number of reviews that have appeared on the topic in recent years [10, 13, 16].

Despite signs that RHC is maturing and formalising as a research paradigm [13, 16, 59], there have been calls for continued developments to strengthen RHC theory and research [13]. As evidenced by this review, the COVID-19 pandemic presented a unique opportunity to research and critically advance our understanding of RHC, and in particular, created a shift in focus from theoretical conceptualisations to identifying how we might understand factors or capacities that foster resilience across the health system [83]. Previously, empirical studies on RHC were rare and skewed towards the clinical microsystems of care, however, the surge of literature on RHC during the pandemic provided a unique opportunity to take stock of the empirical landscape [83]. Indeed, since the previous review by Iflaifel et al. [16], which found 71 empirical studies on RHC over an 18-year period, the present scoping review identified a further 50 studies, highlighting the unprecedented growth of empirical applications within the RHC field over the past three years.

Consistent with previous reviews [13, 16], qualitative methods dominated the included studies, with interviews typically being used to capture healthcare workers’ perceptions and experiences during the pandemic. Although the extensive use of qualitative methods has been cited as one of the strengths of RHC [13], this review saw the application of existing tools (e.g., FRAM, SPLINTS) along with the emergence of new quantitative assessments and indicator-based modelling approaches that could have fruitful implications, particularly in terms of enhancing system preparedness and advancing measurement and monitoring of resilient performance over time. We also identified the development of new questionnaires to assess RHC; many of which were based on a conceptual framework (e.g., such as Hollnagel’s [53] ‘four cornerstones of resilience’ and Anderson et al.’s [55] Integrated Resilience Attributes Framework). In addition, we saw an increased number of studies examining RHC in LMICs. For example, the two studies of Karamaji et al. [48, 49] presented an approach to assessing and monitoring health systems functionality in developing African countries, with a set of indicators that combine into a “resilience index”, each with varying levels of “transformation capacity”. While RHC theorists have historically resisted establishing indicators and measurement in this field, some people are expressing a need to advance our understanding of system resilience beyond the conventional health system building blocks of the WHO published 15 years ago [60]; thus, including measurement and monitoring is increasingly pressing.

A previous criticism has been that a preponderance of studies of RHC at micro and meso levels is “not sufficient to understand systems resilience” [84], and thus it was promising to see the emergence of macro level studies in this review. The macro-level study by Smaggus et al. [14], for example, examined government responses to the pandemic, by way of a document analysis of media releases, in two countries, Canada and Australia, expanding the scope of RHC research to different system levels, and incorporating a cross-country comparison [84]. Furthermore, Smaggus et al. [14] integrated several resilience theoretical frameworks to guide their study, illustrating how theory can inform research design and analysis. However, this study also highlighted some of the difficulties of researching RHC, particularly at the macro level, and that a mixed-methods approach (e.g., including interviews and observations alongside document analysis) would be likely to provide a more complex understanding on how government actions affect health system resilience, and build a better understanding of the links between actions at the macro level and other system levels.

What was clear was that the included studies reported varying degrees of preparedness and adaptive capacity across the different healthcare services. For example, a number of studies reported how well organisations or the people who work in them “evolved” to make things work [39, 54, 81], while others reported extreme physical and emotional demands, leading to stress and burnout amongst healthcare workers and poor clinical care [37, 39, 43, 65, 69, 73]. This discrepancy between resilient performance and physical and emotional burnout could be explained by the extensive use of short-term adaptations, rather than long-term innovation and system change [35]. This tradeoff between short and long term adaptations can also be expressed as a tradeoff between “specified” and “general” resilience [85]. Healthcare personnel initiating short term adaptations and workarounds, such as taking on extra responsibility, working longer shifts, often without breaks to compensate for systems deficiencies, such as workforce shortages, may only have a short-term ‘firefighting’ effect on the specific situation [86]. Without long-term, general adaptations that foster organisational and system change, short term adaptations could potentially end up as a barrier for systemic resilient performance instead of a capacity [55, 87, 88].

This issue also reminds us of Woods [89] notion that all systems have an “envelope of performance”; a range of how much they can adapt, due to finite resources and the inherent variation in the system. When a system is pushed to the edge of its envelope, the system can either adapt and expand its performance further into “graceful extensibility” or become “brittle” and potentially lead to system collapse. Wear and Hettinger [90] also pointed to circumstances where local adaptations may become too extensive (the “tragedy of adaptability”). In the case of COVID-19, the continuous need for short-term adaptations placed the responsibility of the system’s ability for resilient performance on the sharp-end agents rather than the system itself, who over time became physically and emotionally exhausted. Although RHC has not often considered an individual’s coping and resilience capacity, how individual-level resilience interacts with team-, organizational- and broader systems resilience is a key area for future research.

An important contribution of this study is the recognition of eight key factors or capacities in the existing literature that potentially develop and enhance resilient performance. Recognising that healthcare is highly complex and unpredictable, and understanding that these factors were identified from studies in the context of COVID-19, these findings are highly concordant with the “capacities for resilient performance” identified in the qualitative study by Lyng et al. [30]. It is hoped that the capacities identified in this study can be facilitated and supported through the development of tools and interventions [91]. As identified by Lyng et al. [30] there were obvious interdependencies between the capacities; for example, between structure and leadership, given that leaders often facilitated the implementation and adherence to different structural features such as technology, guidelines or learning arenas; and between coordination and learning given that the greatest number of learning efforts related to team training and coordinating efforts to tackle the challenges related to COVID-19.

One noticeable difference, however, between our findings and those reported by Lyng et al., [30] was the emphasis placed of the the need for teamwork and collaboration during COVID-19. While Lyng et al. [30] suggested that different capacities require different levels of collaboration, higher levels of collaboration may have been required across all eight capacities during the pandemic. Again, this may reflect that many of the adaptations reported were largely reactive efforts focused on system recovery and restoring its equilibrium, particularly during the early stages of the pandemic, thus requiring short-term workarounds or solutions particularly at the front lines of care; but which are noble and important responses to handle peak activity situations [87]. Furthermore, COVID-19 prompted higher levels of collaboration, with the need to ‘rally together’ as they faced the same issues or ‘enemy’ across contexts and system levels. In the same way, two capacities presented by Lyng et al., namely ‘facilitators’ by way of champions and ‘competence’ by way of experience and knowledge, were less prominent in the present study. This is not to say that Lyng et al.’s capacities of competence and facilitators are not important for resilient performance, but rather, in the context of the pandemic, that the collaborative efforts needed to adapt to their joint challenges, may have made individual competencies and facilitators less important, or they were not reported in our included studies. Future studies should continue advancing this theoretical framework in order to integrate factors from different countries and settings and under different situations (stress, crisis, ordinary). Arguably, three of the most important capacities in advancing systems from reactive short-term adaptations at the micro-system level to longer-term “graceful extensibility” are effective leadership, communication and learning [92]. Indeed, examples of interventions promoting these three capacities are appearing in the literature [92–94]. For example, ‘tiered team huddles’ to enable sharing of ideas and issues from health workers at the ‘sharp end’ with middle and senior leadership, enabling communication across boundaries and enabling organizational learning [92]. A ‘learning health system’ [95, 96], cultivated through innovative interventions like tiered team huddles, could improve communication across boundaries and facilitate long-term lasting change. Leaders also need to consider the negative impacts of short-term adaptations and workarounds on staff mental health.

The importance of system “slack” (or “buffer”) at an organizational level and collective level (i.e., network or national), was also highlighted in the study findings, to ensure that the healthcare system is prepared and enables organizational flexibility to deploy equipment and staff rapidly and effectively to where they are needed most [97]. The provision of a margin of manoeuvrability may also reduce the resulting negative effects of continuous micro-adaptations and increased staff workloads; thereby serving as a protective [98] mechanism.

### Implications for research, policy and practice

Despite that the literature confirms that resilience-based efforts and analysis need to occur across system levels (i.e., micro, meso, macro), there is still relatively little understanding – both conceptually and empirically – about how the system levels interact with each other. Although the pandemic affected all system levels, presenting the perfect opportunity to study “cross-level interactions”, most of our included studies focused on one level of analysis. Yet as our review showed, there can be a “dark side or downside of resilience” [29]. What started out as resilient short-term adaptations were exhausting for the people working in the system, resulting in stress and burnout. Considerations for how individual-level resilience factors affect resilience factors at the team and organization-level is an important area for future research.

Of course, identifying the interactions between system levels is challenging, given the non-linear nature of such interactions and the time over which they may occur. Again, this issue points to the need for mixed methods (quantitative and qualitative) approaches, the dual consideration of both positive consequences (e.g., performance, efficiency, safety outcomes), and negative consequences (e.g., by including measures of stress, job satisfaction and burnout) of systems resilience, as well as the need to collect data longitudinally to increase our understanding of causal processes between the various system levels. Although quantitative resilience tools are emerging in the literature, more work is needed to establish theory driven and well validated tools for application at the various system levels.

In this study, the resilience capacities developed by Lyng et al. [30] proved to be an applicable and useful framework. Further empirical research building on this framework would be valuable, such as clarifying the degree of interrelatedness between the capacities, as well as designing and testing interventions around the capacities. One issue remains to be resolved, however; clarification is needed as to whether resilience should be studied as an “outcome, mediator, or determinant of a system’s performance” [83]. Some previous studies use these interchangeably: with resilience described as an underlying potential required to achieve a given outcome, while at the same time concluding that the system “was” or “proved” to be resilient. The capacity approach that we have taken here suggests that resilience is an underlying potential of the system, at its various levels, to adapt or restore its functions in response to disruption. We also call on researchers to be specific about whether they are referring to reactive adaptations focused on recovery or proactive efforts to minimise brittleness, with Woods’ [99] four conceptions of resilience potentially serving as a useful framework in this regard.

The results of this study, in combination with the Lyng et al.’s [30] capacities for resilient performance framework, can be used to guide interventions to support, develop or strengthen resilience. Understanding factors that develop or enhance RHC is critical to developing interventions and tools for strengthening their resilience [100]. This study thereby contributes to this work with key insights for intervention development that can be employed to enhance resilience performance.

### Strengths and limitations

Data analysis and synthesis built on and strengthened the work of Lyng et al.’s [30] capacities for resilient performance framework; this framework can be further used as a basis to guide the next wave of research on RHC. The limitations of this review are primarily methodological. Due to our search strategy, we may have not identified valuable findings published in books, research reports and white papers. Future reviews of empirical studies in this field would benefit from by-hand searching particularly of books, where much of the foundational RHC literature has been identified [13]. Although we identified a relatively high proportion of articles from medium-income countries, our restriction to records in English and published works may have underestimated the true amount of literature emerging from LMIC. Our data extraction was also restricted to what was reported and discussed in the included studies. As a result, we may have under identified some important capacities and negative consequences. Using a data-based convergent synthesis approach, we transformed data from quantitative studies into categories or themes and did not analyse or report the results separately for different study types. Future research involving innovative methods for combining systematic review, concept analysis and bibliometric analysis could be used to summarise qualitative, quantitative and mixed methods RHC studies [101].

## Conclusions

Our review identified an explosion of new empirical studies on health system resilience associated with COVID-19. The pandemic provided a unique ‘natural experiment’ and unprecedented opportunity to examine RHC theory in practice, and uncovered emerging new evidence on RHC theory and system factors that contribute to resilient performance at micro, meso and macro levels. Additionally, we identified potential unintended consequences of short-term responses to improve resilience without due consideration of the longer-term effects. These findings will facilitate strengthening of health system performance and resilience in responding to challenges and other unexpected events in the future.

### Electronic supplementary material

Below is the link to the electronic supplementary material.

Supplementary Material 1

Supplementary Material 2

Supplementary Material 3

Supplementary Material 4

## Data Availability

The datasets used and/or analysed during the current study are available from the corresponding author on reasonable request.

## Abbreviations

FRAM: Functional Resonance Analysis Method
PRISMA-ScR: Preferred Reporting Items of Systematic Review and Meta-Analyses Extension for Scoping Reviews
RHC: Resilient Health Care
SPLINTS: Scrub Practitioners List of Intra-operative Non-Technical Skills
WHO: World Health Organisation
LMIC: Low- and middle-income countries

## References

[1] Hollnagel E, Braithwaite J, Wears RL. Preface: on the need for resilience in health care. Resilient health care Farnham: Ashgate Publishing. 2013:2–3.

[2] Wiig S, O’Hara JK (2021). Resilient and responsive healthcare services and systems: challenges and opportunities in a changing world. BMC Health Serv Res.

[3] World Health Organization. Health systems resilience toolkit: a WHO global public health good to support building and strengthening of sustainable health systems resilience in countries with various contexts. 2022.

[4] World Health Organization. Building Health Systems Resilience for Universal Health Coverage and Health Security during the COVID-19 Pandemic and beyond: WHO Position Paper.; 2021.

[5] World Health Organization. Strengthening health emergency preparedness and response in the WHO South-East Asia Region building upon lessons learnt from COVID-19. World Health Organization. Regional Office for South-East Asia; 2022.

[6] World Health Organization. Outbreak preparedness and resilience. 2020.

[7] Ghebreyesus TA, Jakab Z, Ryan MJ, Mahjour J, Dalil S, Chungong S (2022). WHO recommendations for resilient health systems. Bull World Health Organ.

[8] Hollnagel E. About The Resilient Health Care Net [cited 2023 Jan 31] Available from: https://resilienthealthcare.net/about/.

[9] Woods DD, Hollnagel E, Prologue (2017). Resilience engineering concepts.

[10] Fridell M, Edwin S, Von Schreeb J, Saulnier DD (2020). Health system resilience: what are we talking about? A scoping review mapping characteristics and keywords. Int J Health Policy Manage.

[11] Tiernan A, Drennan L, Nalau J, Onyango E, Morrissey L, Mackey B (2019). A review of themes in disaster resilience literature and international practice since 2012. Policy Des Pract.

[12] Barasa E, Mbau R, Gilson L (2018). What is resilience and how can it be nurtured? A systematic review of empirical literature on organizational resilience. Int J Health Policy Manage.

[13] Ellis LA, Churruca K, Clay-Williams R, Pomare C, Austin EE, Long JC (2019). Patterns of resilience: a scoping review and bibliometric analysis of resilient health care. Saf Sci.

[14] Smaggus A, Long JC, Ellis LA, Clay-Williams R, Braithwaite J (2022). Government actions and their relation to resilience in healthcare during the COVID-19 pandemic in New South Wales, Australia and Ontario, Canada. Int J Health Policy Manage.

[15] Biddle L, Wahedi K, Bozorgmehr K (2020). Health system resilience: a literature review of empirical research. Health Policy Plann.

[16] Iflaifel M, Lim RH, Ryan K, Crowley C (2020). Resilient health care: a systematic review of conceptualisations, study methods and factors that develop resilience. BMC Health Serv Res.

[17] Braithwaite J, Churruca K, Ellis LA, Long J, Clay-Williams R, Damen N (2017). Complexity science in healthcare.

[18] Nyssen A-S, Bérastégui P. Is system resilience maintained at the expense of individual resilience. Resilient health care III: reconciling work-as-imagined and work-as-done. 2016:37–47.

[19] Chirico F, Ferrari G, Nucera G, Szarpak L, Crescenzo P, Ilesanmi O (2021). Prevalence of anxiety, depression, burnout syndrome, and mental health disorders among healthcare workers during the COVID-19 pandemic: a rapid umbrella review of systematic reviews. J Health Soc Sci.

[20] Vizheh M, Qorbani M, Arzaghi SM, Muhidin S, Javanmard Z, Esmaeili M (2020). The mental health of healthcare workers in the COVID-19 pandemic: a systematic review. J Diabetes Metabolic Disorders.

[21] Chen W, Hirschheim R (2004). A paradigmatic and methodological examination of information systems research from 1991 to 2001. Inform Syst J.

[22] Tricco AC, Lillie E, Zarin W, O’Brien KK, Colquhoun H, Levac D et al. PRISMA Extension for Scoping Reviews (PRISMA-ScR): Checklist and Explanation. Ann Intern Med [Internet]. 2018; 169(7):[467 – 73 pp.]. Available from: https://www.ncbi.nlm.nih.gov/pubmed/30178033. 10.7326/M18-0850 30178033

[23] Peters MDJ, Marnie C, Tricco AC, Pollock D, Munn Z, Alexander L et al. Updated methodological guidance for the conduct of scoping reviews. JBI Evid Synth [Internet]. 2020; 18(10):[2119-26 pp.]. Available from: https://www.ncbi.nlm.nih.gov/pubmed/33038124. 10.11124/JBIES-20-00167 33038124

[24] Paré G, Trudel M-C, Jaana M, Kitsiou S. Synthesizing information systems knowledge: a typology of literature reviews. Inf Manag [Internet]. 2015; 52(2):[183 – 99 pp.].

[25] Pham MT, Rajic A, Greig JD, Sargeant JM, Papadopoulos A, McEwen SA. A scoping review of scoping reviews: advancing the approach and enhancing the consistency. Res Synth Methods [Internet]. 2014 Dec PMC4491356]; 5(4):[371 – 85 pp.]. Available from: https://www.ncbi.nlm.nih.gov/pubmed/26052958. 10.1002/jrsm.1123 PMC4491356 26052958

[26] Hong QN, Pluye P, Bujold M, Wassef M (2017). Convergent and sequential synthesis designs: implications for conducting and reporting systematic reviews of qualitative and quantitative evidence. Syst Reviews.

[27] Sandelowski M (1991). Telling stories: narrative approaches in qualitative research. Image: The Journal of Nursing Scholarship.

[28] The World Bank. World Bank Country and Lending Groups: The World Bank. ; 2021 [Available from: https://datahelpdesk.worldbank.org/knowledgebase/articles/906519-world-bank-country-and-lending-groups.

[29] Thomas J, Harden A (2008). Methods for the thematic synthesis of qualitative research in systematic reviews. BMC Med Res Methodol.

[30] Lyng HB, Macrae C, Guise V, Haraldseid-Driftland C, Fagerdal B, Schibevaag L (2022). Capacities for resilience in healthcare; a qualitative study across different healthcare contexts. BMC Health Serv Res.

[31] Alajmi A, Adlan N, Lahyani R (2021). Assessment of Supply Chain Management Resilience within Saudi Medical Laboratories during Covid-19 pandemic. Procedia Cirp.

[32] Smith EM, Hernandez MLT, Ebuenyi I, Syurina EV, Barbareschi G, Best KL et al. Assistive technology use and provision during COVID-19: results from a rapid global survey. Int J Health Policy Manage. 2020. 10.34172/ijhpm.2020.210 PMC9309903 33201656

[33] Montás MC, Klasa K, van Ginneken E, Greer SL. Strategic purchasing and health systems resilience: Lessons from COVID-19 in selected european countries. Health Policy. 2022. 10.1016/j.healthpol.2022.06.005 PMC9195347 35773063

[34] Spieske A, Gebhardt M, Kopyto M, Birkel H. Improving resilience of the healthcare supply chain in a pandemic: evidence from Europe during the COVID-19 crisis. J Purchasing Supply Manage. 2022:100748.

[35] Lyng HB, Ree E, Wibe T, Wiig S (2021). Healthcare leaders’ use of innovative solutions to ensure resilience in healthcare during the Covid-19 pandemic: a qualitative study in norwegian nursing homes and home care services. BMC Health Serv Res.

[36] Marshall F, Gordon A, Gladman JR, Bishop S (2021). Care homes, their communities, and resilience in the face of the COVID-19 pandemic: interim findings from a qualitative study. BMC Geriatr.

[37] Martin A, Hatzidimitriadou E (2022). Optimising health system capacity: a case study of community care staff’s role transition in response to the coronavirus pandemic. Health Soc Care Commun.

[38] Cannedy S, Bergman A, Medich M, Rose DE, Stockdale SE (2022). Health System Resiliency and the COVID-19 pandemic: a case study of a New Nationwide Contingency staffing program.

[39] Brenner AB, Knaub M, Robinson K, Lotspeich M, Eisen J. Building Resilience in the Face of Crisis: Lessons learned from a community behavioral Healthcare Organization. J Behav Health Serv Res. 2022:1–8. 10.1007/s11414-021-09781-1 PMC8743071 35001257

[40] van Gool F, Bongers I, Bierbooms J, Janssen R. Whether and how top management create flexibility in mental healthcare organizations: COVID-19 as a test case. J Health Organ Manag. 2022. 10.1108/JHOM-07-2021-0258 35238189

[41] Danhieux K, Buffel V, Pairon A, Benkheil A, Remmen R, Wouters E (2020). The impact of COVID-19 on chronic care according to providers: a qualitative study among primary care practices in Belgium. BMC Fam Pract.

[42] Leslie M, Fadaak R, Pinto N, Davies J, Green L, Seidel J (2021). Achieving resilience in primary care during the COVID-19 pandemic: competing visions and lessons from Alberta. Healthc Policy.

[43] Lotta G, Fernandez M, Corrêa M (2021). The vulnerabilities of the brazilian health workforce during health emergencies: analysing personal feelings, access to resources and work dynamics during the COVID-19 pandemic. Int J Health Plann Manag.

[44] Yoon S, Goh H, Chan A, Malhotra R, Visaria A, Matchar D (2022). Spillover effects of COVID-19 on essential chronic care and ways to foster health system resilience to support vulnerable non-COVID patients: a multistakeholder study. J Am Med Dir Assoc.

[45] Juárez-Ramírez C, Reyes-Morales H, Gutiérrez-Alba G, Reartes-Peñafiel DL, Flores-Hernández S, Muños-Hernández JA (2022). Local health systems resilience in managing the COVID-19 pandemic: lessons from Mexico. Health Policy Plann.

[46] Stengel S, Roth C, Breckner A, Cordes L, Weber S, Ullrich C (2022). Resilience of the primary health care system–german primary care practitioners’ perspectives during the early COVID-19 pandemic. BMC Prim Care.

[47] Jovanović A, Klimek P, Renn O, Schneider R, Øien K, Brown J (2020). Assessing resilience of healthcare infrastructure exposed to COVID-19: emerging risks, resilience indicators, interdependencies and international standards. Environ Syst Decisions.

[48] Karamagi HC, Titi-Ofei R, Kipruto HK, Seydi AB-W, Droti B, Talisuna A (2022). On the resilience of health systems: a methodological exploration across countries in the WHO African Region. PLoS ONE.

[49] Karamagi HC, Tumusiime P, Titi-Ofei R, Droti B, Kipruto H, Nabyonga-Orem J (2021). Towards universal health coverage in the WHO African Region: assessing health system functionality, incorporating lessons from COVID-19. BMJ Global Health.

[50] Hollnagel E. FRAM: the functional resonance analysis method: modelling complex socio-technical systems. Crc Press; 2017.

[51] Malec JF, Torsher LC, Dunn WF, Wiegmann DA, Arnold JJ, Brown DA (2007). The mayo high performance teamwork scale: reliability and validity for evaluating key crew resource management skills. Simul Healthc.

[52] Mitchell L (2017). Scrub practitioners’ list of intra-operative non-technical skills–SPLINTS.

[53] Hollnagel E. The four cornerstones of resilience engineering. Resilience Engineering Perspectives. Volume 2. CRC Press; 2016. pp. 139–56.

[54] Corbaz-Kurth S, Juvet TM, Benzakour L, Cereghetti S, Fournier C-A, Moullec G (2022). How things changed during the COVID-19 pandemic’s first year: a longitudinal, mixed-methods study of organisational resilience processes among healthcare workers. Saf Sci.

[55] Anderson J, Ross A, Macrae C, Wiig S (2020). Defining adaptive capacity in healthcare: a new framework for researching resilient performance. Appl Ergon.

[56] Juvet TM, Corbaz-Kurth S, Roos P, Benzakour L, Cereghetti S, Moullec G (2021). Adapting to the unexpected: problematic work situations and resilience strategies in healthcare institutions during the COVID-19 pandemic’s first wave. Saf Sci.

[57] Bueno WP, Saurin TA, Wachs P, Kuchenbecker R, Braithwaite J (2019). Coping with complexity in intensive care units: a systematic literature review of improvement interventions. Saf Sci.

[58] Saurin TA, Wachs P, Bueno WP, de Souza Kuchenbecker R, Boniatti MM, Zani CM (2022). Coping with complexity in the COVID pandemic: an exploratory study of intensive care units. Hum Factors Ergon Manuf Serv Ind.

[59] Macrae C, Wiig S. Resilience: from practice to theory and Back again. Exploring resilience a scientific journey from practice to theory Cham: Springer Open. 2019:121-8.

[60] World Health Organization. Everybody’s business–strengthening health systems to improve health outcomes: WHO’s framework for action. 2007.

[61] Seruwagi G, Nakidde C, Otieno F, Kayiwa J, Luswata B, Lugada E (2021). Healthworker preparedness for COVID-19 management and implementation experiences: a mixed methods study in Uganda’s refugee-hosting districts. Confl Health.

[62] Balqis-Ali NZ, Fun WH, Ismail M, Ng RJ, Jaaffar FSA, Low LL (2021). Addressing gaps for Health Systems strengthening: a public perspective on Health Systems’ response towards COVID-19. Int J Environ Res Public Health.

[63] Khalil M, Mataria A, Ravaghi H (2022). Building resilient hospitals in the Eastern Mediterranean Region: lessons from the COVID-19 pandemic. BMJ Global Health.

[64] World Health Organization. Hospital readiness checklist for COVID-19 interim version February 24 2020. World Health Organization. Regional Office for Europe; 2020.

[65] Ballantyne H, Achour N. The challenges of nurse redeployment and opportunities for leadership during COVID-19 pandemic. Disaster Med Pub Health Prep. 2022:1–7. 10.1017/dmp.2022.43 PMC8961057 35152933

[66] Gifford R, Fleuren B, van de Baan F, Ruwaard D, Poesen L, Zijlstra F (2022). To uncertainty and Beyond: identifying the Capabilities needed by hospitals to function in dynamic environments. Med Care Res Rev.

[67] Parikh N, Chaudhuri A, Syam SB, Singh P. Fostering resilient Health Systems in India: Providing Care for PLHIV under the Shadow of COVID-19. Front Public Health. 2022:1581. 10.3389/fpubh.2022.836044 PMC9194090 35712311

[68] Shah A, Guessi M, Wali S, Ware P, McDonald M, O’Sullivan M (2021). The resilience of cardiac care through virtualized services during the COVID-19 pandemic: case study of a heart function clinic. JMIR cardio.

[69] Abu Mansour SI, Abu Shosha GM (2022). Experiences of first-line nurse managers during COVID‐19: a jordanian qualitative study. J Nurs Adm Manag.

[70] Graetz DE, Sniderman E, Villegas CA, Kaye EC, Ragab I, Laptsevich A (2022). Resilient health care in global pediatric oncology during the COVID-19 pandemic. Cancer.

[71] Hodgins M, Van Leeuwen D, Braithwaite J, Hanefeld J, Wolfe I, Lau C (2022). The COVID-19 system shock framework: capturing health system innovation during the COVID-19 pandemic. Int J Health Policy Manage.

[72] Aliyu S, Norful AA, Schroeder K, Odlum M, Glica B, Travers JL (2021). The powder keg: Lessons learned about clinical staff preparedness during the early phase of the COVID-19 pandemic. Am J Infect Control.

[73] Britton CR, Hayman G, Stroud N (2021). Awareness of human factors in the operating theatres during the COVID-19 pandemic. J Perioper Pract.

[74] Dunleavy L, Preston N, Bajwah S, Bradshaw A, Cripps R, Fraser LK (2021). Necessity is the mother of invention’: specialist palliative care service innovation and practice change in response to COVID-19. Results from a multinational survey (CovPall). Palliat Med.

[75] Lakissian Z, Sabouneh R, Zeineddine R, Fayad J, Banat R, Sharara-Chami R (2020). In-situ simulations for COVID-19: a safety II approach towards resilient performance. Adv Simul.

[76] Nair S, Kannan P, Mehta K, Raju A, Mathew J, Ramachandran P (2021). The COVID-19 pandemic and its impact on mental health services: the provider perspective. J Public Health.

[77] Graetz DE, Sniderman E, Villegas C, Ragab I, Laptsevich A, Maliti B (2022). Utilizing multilingual methods and Rapid Analysis for Global qualitative research during a pandemic. Global Qualitative Nursing Research.

[78] Snowdon A, Wright A, editors. Digitally enabled supply chain as a strategic asset for the COVID-19 response in Alberta. Healthcare Management Forum; 2022: SAGE Publications Sage CA: Los Angeles, CA. 10.1177/08404704211057525 PMC8841392 35144506

[79] Peat G, Olaniyan J, Fylan B, Breen L, Grindey C, Hague I, et al. Mapping the resilience performance of community pharmacy to maintain patient safety during the Covid-19 pandemic. Research in Social and Administrative Pharmacy; 2022. 10.1016/j.sapharm.2022.01.004 PMC8762922 35082103

[80] Akinyemi OO, Popoola OA, Fowotade A, Adekanmbi O, Cadmus EO, Adebayo A (2021). Qualitative exploration of health system response to COVID-19 pandemic applying the WHO health systems framework: case study of a nigerian state. Sci Afr.

[81] McCollum R, Zaizay Z, Dean L, Watson V, Frith L, Alhassan Y (2022). Qualitative study exploring lessons from Liberia and the UK for building a people-centred resilient health systems response to COVID-19. BMJ Open.

[82] Pilevari N, Shiva MV (2021). Country-wide resilience model for the health system: a case study on iran, under coronavirus outbreak. Iran J Public Health.

[83] Bozorgmehr K, Zick A, Hecker T (2022). Resilience of Health Systems: understanding uncertainty uses, intersecting crises and cross-level interactions; comment on “Government actions and their relation to Resilience in Healthcare during the COVID-19 pandemic in New South Wales, Australia and Ontario, Canada. Int J Health Policy Manage.

[84] Anderson JE, Theorising Health System Resilience and the Role of Government Policy-Challenges and Future Directions (2022). Comment on “Government actions and their relation to Resilience in Healthcare during the COVID-19 pandemic in New South Wales, Australia and Ontario, Canada. Int J Health Policy Manage.

[85] Walker B, Westley F. Perspectives on resilience to disasters across sectors and cultures. Ecol Soc. 2011;16(2).

[86] Löf A (2010). Exploring adaptability through learning layers and learning loops. Environ Educ Res.

[87] Lyng HB, Macrae C, Guise V, Haraldseid-Driftland C, Fagerdal B, Schibevaag L (2021). Balancing adaptation and innovation for resilience in healthcare–a metasynthesis of narratives. BMC Health Serv Res.

[88] Glette MK, Wiig S (2021). The role of organizational factors in how efficiency-thoroughness trade-offs potentially affect clinical quality dimensions–a review of the literature. Int J Health Gov.

[89] Woods DD. Resilience as Graceful Extensibility to Overcome Brittleness i. An edited collection of authored pieces comparing, contrasting, and integrating risk and resilience with an emphasis on ways to measure resilience. 2016:258.

[90] Wears RL, Hettinger AZ (2014). The tragedy of adaptability. Ann Emerg Med.

[91] Ree E, Haraldseid-Driftland C. How can Digital Learning Tools be used to promote resilience in Healthcare? Resilience in a Digital Age: Global Challenges in Organisations and Society. Springer; 2022. pp. 231–45.

[92] Rangachari P, Woods L (2020). Preserving organizational resilience, patient safety, and staff retention during COVID-19 requires a holistic consideration of the psychological safety of healthcare workers. Int J Environ Res Public Health.

[93] Wahl K, Stenmarker M, Ros A (2022). Experience of learning from everyday work in daily safety huddles—a multi-method study. BMC Health Serv Res.

[94] Ree E, Ellis LA, Wiig S (2021). Managers’ role in supporting resilience in healthcare: a proposed model of how managers contribute to a healthcare system’s overall resilience. Int J Health Gov.

[95] Ellis LA, Sarkies M, Churruca K, Dammery G, Meulenbroeks I, Smith CL (2022). The science of learning health systems: scoping review of empirical research. JMIR Med Inf.

[96] Pomare C, Mahmoud Z, Vedovi A, Ellis LA, Knaggs G, Smith CL (2022). Learning health systems: a review of key topic areas and bibliometric trends. Learn Health Syst.

[97] McHugh JP, Cross DA (2021). The application of organizational slack to hospital system responsiveness during the COVID-19 pandemic. J Hosp Manage Health Policy.

[98] Saurin TA, editor. Removing waste while preserving slack: the lean and complexity perspectives. LC3 2017 Volume II–Proceedings of the 25th Annual Conference of the International Group for Lean, Heraklion, Greece; 2017.

[99] Woods DD (2015). Four concepts for resilience and the implications for the future of resilience engineering. Reliab Eng Syst Saf.

[100] Wiig S, O’Hara JK (2021). Resilient and responsive healthcare services and systems: challenges and opportunities in a changing world. BMC Health Serv Res.

[101] Gartner J-B, Abasse KS, Bergeron F, Landa P, Lemaire C, Côté A (2022). Definition and conceptualization of the patient-centered care pathway, a proposed integrative framework for consensus: a Concept analysis and systematic review. BMC Health Serv Res.

