# Supplementary material for: The rise of resilient healthcare research during COVID-19: scoping review of empirical research
Source: BMC Health Serv Res. 2023 Aug 7;23:833. doi: 10.1186/s12913-023-09839-0 (PMC10405417; doi:10.1186/s12913-023-09839-0)
Supplement: Supplementary file 2 — Supplementary Material 2 [file 12913_2023_9839_MOESM2_ESM.docx]

**Supplementary File 2: Database Search Strategy**

*Database search strategy: Ovid Medline, EMBASE, Scopus, and Safety Science abstracts in ProQuest*

| Number | Search terms |
| --- | --- |
| 1 | (resili* or resilient healthcare or resilient health care or safety I or safety II or safety 1 or safety 2 or work as imagined or work as done).mp. |
| 2 | (healthcare OR health care OR health-care OR hospital* OR health facilit* OR acute care OR health organi* OR health system* OR primary care OR primary health* OR community health* OR general practice OR aged care).mp |
| 3 | Delivery of Health Care/ |
| 4 | OR/2-3 |
| 5 | (emotional resili* OR personal resili* OR individual resili* OR carer resili* OR professional resili* OR psycho* resili* OR family resili*).mp |
| 6 | Resilience, Psychological/ |
| 7 | OR/5-6 |
| 8 | AND/1,4 |
| 9 | 8 NOT 7 |
| 10 | (((exp Coronavirus/ or exp Coronavirus Infections/ or (coronavirus* or corona virus* or OC43 or NL63 or 229E or HKU1 or HCoV* or ncov* or covid* or sars-cov* or sarscov* or Sars-coronavirus* or Severe Acute Respiratory Syndrome Coronavirus*).mp.) and 20190601:20301231.(ep).) not (SARS or SARS-CoV or MERS or MERS-CoV or Middle East respiratory syndrome or camel* or dromedar* or equine or coronary or coronal or covidence* or covidien or influenza virus or HIV or bovine or calves or TGEV or feline or porcine or BCoV or PED or PEDV or PDCoV or FIPV or FCoV or SADS-CoV or canine or CCov or zoonotic or avian influenza or H1N1 or H5N1 or H5N6 or IBV or murine corona*).mp.) or Covid-19/ or (covid or covid19 or 2019-ncov or ncov19 or ncov-19 or 2019-novel CoV or sars-cov2 or sars-cov-2 or sarscov2 or sarscov-2 or Sars-coronavirus2 or Sars-coronavirus-2 or SARS-like coronavirus* or coronavirus-19 or ((novel or new or nouveau) adj2 (CoV or nCoV or covid or coronavirus* or corona virus or Pandemi*2)) or (variant* adj2 (India* or "South Africa*" or UK or English or Brazil* or alpha or beta or delta or gamma or kappa or lambda or "P.1" or "C.37")) or ("B.1.1.7" or "B.1.351" or "B.1.617.1" or "B.1.617.2or Omnicron")) |
| 11 | AND/9-10 |
| 12 | limit 11 to (english language and yr="2020 -Current") |

Note. * symbolises truncation
