# Supplementary material for: The rise of resilient healthcare research during COVID-19: scoping review of empirical research
Source: BMC Health Serv Res. 2023 Aug 7;23:833. doi: 10.1186/s12913-023-09839-0 (PMC10405417; doi:10.1186/s12913-023-09839-0)
Supplement: Supplementary file 4 — Supplementary Material 4 [file 12913_2023_9839_MOESM4_ESM.docx]

| **Supplementary File 4. Summary themes and subthemes** | | | |
| --- | --- | --- | --- |
| Themes | Sub-themes | n | Examples |
| Coordination | Team cohesion | 10 | “Peer support…the exchange of experiences between colleagues, reassurance, emotionally supporting each other while dealing with the same work stressors, cooperative teamwork, and effective communication” (1)  “Clinicians expressed a sense of comradery and worked together toward a common goal” (2)  “Many participants expressed a greater feeling of teamwork. Hierarchies seemed to compress with a leveling of the traditional roles played by different professions. As participants gained experience in caring for COVID-19 patients and as leadership disseminated information, participants’ confidence in their capacity to provide care increase.” (3)  “Really supportive. Rallied together.” (4) |
|  | Multidisciplinary teamwork | 10 | “Team-based coordination regardless of discipline” (3)  “Different professionals… worked together, held multidisciplinary discussions and formed mixed team” (5)  “The ICC includes a particularly diverse group of stakeholders representing each department (clinical and non-clinical). Staff was selected for specific knowledge bases and skill sets necessary for rapid development and implementation of plans, and not based on organizational hierarchy.” (6)  “Using Multidisciplinary teams – importance of multidisciplinary teams working together to improve hospital resilience.” (7) |
|  | Team communication | 5 | “Bidirectional communications that the groups’ members experienced” (8)  “Staff provided feedback to their managers” (6)  “It was used to facilitate communication within specialist palliative care teams, across specialist palliative care services and with generalist clinicians and external partner organisations. Respondents felt the benefits included…keeping the team connected and up to date with the ever-changing situation as well as helping to facilitate the support process” (9)  “Demonstrate effective teamwork behavior by practicing key crisis resource management elements including closed-loop clear communication” (10) |
|  | Inter-organisational coordination | 15 | “hospital emergency team and the clinical staff worked closely together” (3)  “Strategic and timely coordination was the catalyst for resource optimisation and workload distribution” (11)  “As processes and workflows evolved, teamwork evolved as well…There's an attending backup that is actively helping – not just waiting around for work – but actively helping with new COVID-19 e-consults.” (3)  “In particular, resilient health care was fostered by experienced teams who reacted to evolving information and worked together within larger health care systems.” (2)  “In the second wave, some organizations expanded or altered the crisis structure to include formerly underrepresented groups such as nurses and medical specialists, and to give more responsibility to departmental level and line management” (12) |
|  | Intra-organisational coordination | 7 | “The strategy implemented to face these challenges is to collaborate with other institutions that have an emergency supply shortage…enlarge their supplier base”(13)  “Informal networks of companies and voluntary organizations from the local community often supplies resources. Networks with other regional health providers were also critical in dealing with clinical resource and knowledge issues.” (14)  “the hospital case companies buffered dependencies by opening up alternative sources, received more reliable delivery promises, avoided opportunistic behavior of distributors, and sped up procurement processes.” (15)  “Collaborative teamwork (within and between specialist palliative care services and with other generalist palliative care providers).” (9) |
| Leadership | Transparent and open communication | 4 | “The amount and frequency of information coming from leadership increased, and many participants reported that such information increased their perceived ability to care for patients with COVID-19” (3)  “Clear and frequent communication, and leadership, was the foundation of Cascadia’s success in managing the crisis” (6)  “Leaders distributed the continuous ” (16)  “Clear communication and ability to motivate overworked and burnt-out staff are necessary attributes in outbreak response” (7) |
|  | Visibility | 8 | “structured and visible leadership” (17)  “leaders altered their daily work schedules to be present at the sharp end and make sure their staff wore their protection equipment appropriately” (16)  “Strong leadership with clear vision to push through crisis to recovery…” (7)  “Several managers devised “COVID-19 command centres” which met daily to review emerging evidence and data, and devise new care practices” (14) |
|  | Supportive and empowering | 8 | “motivations and rewards, appreciation of hard work, providing logistical support from their hospital, shared decision-making” (1)  “Residential leadership discussed the importance of reaching out to front line staff often between meetings, and with genuine concern for staff’s mental and emotional health” (6)  “Managers… provide reassurance to their staff, many of whom were frightened and exhausted” (14)  “Management support was appreciated, whether relational or organisational. One employee recalled that “the morning meeting started by checking in with everyone, leaving time for questions and the expression of any fears. All topics, even private ones, could be discussed.”” (5) |
|  | Decisive leadership | 10 | “Clear chain of command” (1)  “Leadership took proactive steps to combat the pandemic” (18)  “Leadership staff approached policy making in a decisive, yet flexible way” (6)  “managers reported consciously adapting their leadership style towards a more hierarchical “military” style of command.”(14)  “rapid decision-making processes and rapid activation of experienced and trained crisis teams that dealt with the pandemic on a directional and structural level” (4) |
| Alignment | Role evolution | 13 | “Innovative evolution of roles and responsibilities” (3)  “Many nursing staff were rapidly retrained and redeployed” (17)  “Unusual activities forced employees to adapt to new tasks and to work in other wards and units”(5)  “redeployment of HCPs from different specialties to COVID-19 response duties” (19) |
|  | Workarounds / trade-offs | 19 | “Re-organization of the practice in line with the hygienic guidelines” (20)  “Organisations repurposed wards, redeployed staff, transitioned staff to work from home, and delayed patient care.” (12)  “Learned to manage with fewer resources and maximize what they had” (2)  “Spaces and units were reorganised, and some employees had to work on a different site”(5)  “The masks children wore were decorated with toys, shapes and colors…Now everyone wears the mask, and it is familiar.”(2)  “…innovative solutions for outdoor concerts and shows (like ballet), where the patients could watch from their balconies” (16) |
|  | Restructuring / compensation | 19 | “To compensate for shortages, several strategies were adopted by the hospitals, such as recruiting nurses, establishing field hospitals, and opening new units for COVID-19” (1)  “Organizations had to scale up capacity rapidly for COVID-19 care, in particular by increasing ICU and Emergency care (A&E) capacity” (12)  “The COVID-19 ward management teams developed standard operating procedures (SOPs) that directed practice in response to NSW Health and organisational directives.”(21)  “Participants reported modifying the emergency plan, developing new policies, increasing medical supplies and PPE, and improving safety standards to deal with COVID-19 and strengthen the system and resilience” (1) |
| Learning | On-the-job learning | 9 | “Exposure to a new experience like covid-19 developed their management skills and leadership competencies, such as decision making and communication” (1)  “Trial-and-error approach…capacity building…evolving practical knowledge…on-going daily training” (3)  “Nurses perceive redeployment as an opportunity for positive career development” (17)  “high proportion of HCWs reporting the need to learn new things at work” (22) |
|  | Training | 15 | “Staff were trained for new functions and COVID care, such as performing intubations and working with ventilators and respiratory equipment…Training took place formally at in-house-learning centers or academies, via daylong or multiple day training sessions, or via on-the-job training” (12)  “Training was quickly set up, including courses, simulation workshops or e-learning on specific care practices or equipment” (5)  “training of facility management, workers or their representatives on management of COVID at the facility” (22)  “infectious disease training for staff, including cleaning staff…PPE training modules for donning and doffing PPE” (21)  they trained non-CRH providers how to deliver virtual care, thereby increasing capacity for treating patients when clinics were closed to in-office visits” (23) |
|  | Simulation | 3 | “Simulation team was developed to run weekly interdisciplinary COVID-19 simulations involving deteriorating patients…” (21)  “Simulating such a rare clinical crisis has proven to be highly effective in raising our participants’ confidence and improving their clinical and non-technical skills” (10)  “…during simulation exercises we bring in real life scenarios of an actual case of COVID-19 just to build their capacity, and make sure that they are ready to respond incase we get a confirmed case in the settlement here” (22) |
| Communication systems | Formal communication systems | 10 | “The daily COVID-19 update was posed by communications and executive team members to manage the rapid influx of information surrounding COVID-19” (21)  “Use of electronic communication systems using internal platforms was a pivotal support system for maintaining care delivery, ensuring timely communication, and tracking changing policies” (3)  “Information was transmitted via regular emails, institutions’ intranet, COVID-19 helplines for patient follow-up and meetings on the disease and the protective measures or protocols to be applied.” (5)  “Flow of information from national and regional authorities to the front-line staff through various channels: email, SMS, printed forms in staff rooms, and the Workplace platform” (16) |
|  | Informal communication systems | 10 | “Heard via email, telephone, or other sources, which incorporated being told by peers, gossip and rumor, WhatsApp messages, and instruction on arrival for their usual role” (17)  “Some teams had “a WhatsApp group to share serious or fun information” (5)  “Informal communication channels like WhatsApp groups, which proved useful for decision making, emotional support and load sharing.” (24)  “Communication took place in the team at the practice level, in the team between OIC, in WhatsApp groups, and also by learning about the experiences of others.” (25) |
| Involvement | Communication with patients and families | 14 | “Rostering system that linked patients and the providers effectively either physically or through teleconsultation and ensuring services to a 24-hour helpline for the general public” (24)  “In some locations, volunteer networks (China) or patient navigators (Philippines) helped providers connect with families” (2)  “The SCHN social work department implemented a wellbeing check in via telephone into the isolation rooms to address this challenge, which was reported to improve patient and carer wellbeing according to ward nursing unit managers and medical officers” (21)  “Teleconsultation was another important service delivery adaptation reported by the majority of the respondents. This included reaching out to patients to provide a consultation for acute and chronic illness, psychosocial support as well as to facilitate appropriate care from nearby centers to which they were referred.” (26)  “Ongoing telephone support would also be provided for those patients who were unable to attend outpatients or day therapy services because of restrictions.” (9) |
|  | Meeting patients needs | 10 | “Several RNs and nursing administrators also reported adapting nursing-specific care delivery processes to meet the immediate needs of the patients” (3)  “Maintaining patient access to care during the pandemic” (23)  “Participants unequivocally maintained the need for holistic care of non-COVID patients, including provision of psychosocial and community services through various medium during public health emergencies” (19)  “Services wanted to reassure patients and family carers that ‘the care is still here, it just looks different’” (9) |
| Structure | Financial resources | 5 | “Adequacy, effectiveness and transparency of funds” (18)  “To support the response to COVID-19 the NSW state government established a COVID fund for health services. COVID-19 funding priorities within the Network included assessment clinics, PPE, cleaning, concierge, and the Clinical Communications program, Medtasker” (21)  “This funding enabled many of the innovations that occurred within the Network and proved a workaround for gaps in funding for necessary services”(21)  “financial difficulties in the face of extra running costs for staffing required to cover sick leave, topping up wages to supplement the government furlough scheme for non-essential staff, and paying staff during shielding or self-isolation due to quarantine”(14) |
|  | Physical equipment | 18 | “Hospitals needed to increase the level of PPE, install plastic shields and protections, and source additional medical equipment and supplies”(12)  “One of the main issues was provision of personal protective equipment (PPE). During the early phases of the pandemic, this was in short supply” (14)  “To match increased capacity and COVID demand, hospitals needed to increase the level of PPE, install plastic shields and protections, and source additional medical equipment and supplies (e.g., plastics for the lab). For example, some hospitals rented additional CT scanners for the COVID screening stations and overflow wards.”(12)  “Alberta had the advantage of accurate utilization data, described as the “burn rate,” which informed preparations on managing product shortages, particularly PPE.” (27)  “PPE was supplied from the state level. For nursing personnel and health promoters, the equipment received consisted of masks and disinfectant products. Doctors, due to close contact with patients, received gowns, goggles or face shields. As cases increased, more PPE was needed” (28) |
|  | Workforce | 11 | “Recruitment team expanded their reach in hiring” (6)  “A central issue in the crisis response was recruiting enough staff to cover the increased demand of COVID care and other emergent demands as the crisis continued” (12)  “Under-staffing was attributed to absences due to illness, work stoppages and reassignments to other units” (5)  “To overcome these challenges, HMs recounted recruiting surge staff through volunteering, task-shifting, international recruitment or special contracts.” (7) |
|  | Technology | 27 | “Teleconsultation provided primary care practices with a potential tool to monitor and support patients with chronic diseases from a distance” (20)  “Transition to E-health, video and phone consultations, and in some organizations the repurposing of wards or outpatient clinics to “digital” wards.” (12)  “Operations shifting toward virtual care delivery” (8)  “The Covid-19 pandemic has facilitated the deployment of telemedicine in France. Indeed, budgetary, and regulatory constraints, which existed until then, have been amended to allow the rapid implementation of teleconsultation, tele-expertise and telemonitoring activities in Public Assistance of Paris Hospitals.” (29)  “increased telemedicine, with some respondents raising access-related equity concerns, particularly for elderly populations, who may struggle to engage with telemedicine” (30) |
|  | Governance systems | 19 | “Modifying the emergency plan, developing new policies…improving safety standards” (1)  “Creation or adaptation of existing policy to enable hospitals to respond efficiently to the emergent situation”(12)  “The rapidly evolving knowledge about resources needed to prevent transmission and immediate availability of equipment and protocols” (3)  “emergency management plans, communication, and governance structures were well established in Alberta,” (27)  “The development of new protocols for the sterilization and reuse of PPE, such as face masks” (31) |
| Risk awareness | Emergency preparedness | 10 | “Creation of pandemic preparedness committee with representatives of all clinical disciplines” (3)  “In the midst of the pandemic Cascadia is fine-tuning their emergency preparedness plan” (6)  “Alberta had previous experience with emergencies, such as wildfires (e.g., Fort McMurray) and severe floods (Calgary), which meant that emergency management plans, communication, and governance structures were well established in Alberta”(27)  “In the onset of crisis, hospitals felt it was easy to switch into crisis mode. The need for a crisis structure was apparent, and organizations perceived it as a key enabler of being able to take quick decisive action”(12)  “A vital element of SCHN’s response to the COVID-19 pandemic was a consolidated effort to coordinate and disseminate important information, which has been shown to be vital for emergency preparedness”(21) |
|  | Preparation / proactive | 16 | “Developed key monitoring tools including a PPE dashboard that indicated the levels of PPE stores available, a patient flow dashboard to track individuals who tested positive to COVID-19 journey through the hospital, a literature repository and summary that added COVID-19 clinical literature consistently and an emergency response matrix”(21)  “Monitor international media stations early in the pandemic. This enabled them to gauge the seriousness of the situation daily” (14)  “Managers devised “COVID-19 command centres” which met daily to review emerging evidence and data, and devise new care practices” (14)  “The health facilities had an active taskforce to manage COVID-19 (82%); the facility had a monitoring and evaluation mechanism of the COVID-19 prevention strategies and plans (81%).” (22) |

Note: HCP=Health care professional; HCW=Health care worker; HM=Hospital managers; ICC= Incident Command Center; NSW=New South Wales; OIC=Outpatient infection centers; PPE=Personal protective equipment; RN=Registered nurses; SCHN= Sydney Children’s Hospitals Network

**References**

1. Abu Mansour SI, Abu Shosha GM. Experiences of first‐line nurse managers during COVID‐19: A Jordanian qualitative study. Journal of Nursing Management. 2022;30(2):384-92.

2.Graetz DE, Sniderman E, Villegas CA, Kaye EC, Ragab I, Laptsevich A, et al. Resilient health care in global pediatric oncology during the COVID‐19 pandemic. Cancer. 2022;128(4):797-807.

3. Aliyu S, Norful AA, Schroeder K, Odlum M, Glica B, Travers JL. The powder keg: Lessons learned about clinical staff preparedness during the early phase of the COVID-19 pandemic. American Journal of Infection Control. 2021;49(4):478-83.

4. van Gool F, Bongers I, Bierbooms J, Janssen R. Whether and how top management create flexibility in mental healthcare organizations: COVID-19 as a test case. Journal of Health Organization and Management. 2022;36(5):604-16.

5. Juvet TM, Corbaz-Kurth S, Roos P, Benzakour L, Cereghetti S, Moullec G, et al. Adapting to the unexpected: Problematic work situations and resilience strategies in healthcare institutions during the COVID-19 pandemic’s first wave. Safety Science. 2021;139:105277.

6. Brenner AB, Knaub M, Robinson K, Lotspeich M, Eisen J. Building Resilience in the Face of Crisis: Lessons Learned from a Community Behavioral Healthcare Organization. The Journal of Behavioral Health Services & Research. 2022:1-8.

7. Khalil M, Mataria A, Ravaghi H. Building resilient hospitals in the Eastern Mediterranean Region: lessons from the COVID-19 pandemic. BMJ Global Health. 2022;7(Suppl 3):e008754.

8. Leslie M, Fadaak R, Pinto N, Davies J, Green L, Seidel J, et al. Achieving resilience in primary care during the COVID-19 pandemic: competing visions and lessons from Alberta. Healthcare Policy. 2021;17(2):54.

9. Dunleavy L, Preston N, Bajwah S, Bradshaw A, Cripps R, Fraser LK, et al. ‘Necessity is the mother of invention’: Specialist palliative care service innovation and practice change in response to COVID-19. Results from a multinational survey (CovPall). Palliative Medicine. 2021;35(5):814-29.

10. Lakissian Z, Sabouneh R, Zeineddine R, Fayad J, Banat R, Sharara-Chami R. In-situ simulations for COVID-19: a safety II approach towards resilient performance. Advances in Simulation. 2020;5(1):1-10.

11. Balqis-Ali NZ, Fun WH, Ismail M, Ng RJ, Jaaffar FSA, Low LL. Addressing Gaps for Health Systems Strengthening: A Public Perspective on Health Systems’ Response towards COVID-19. International Journal of Environmental Research and Public Health. 2021;18(17):9047.

12. Gifford R, Fleuren B, van de Baan F, Ruwaard D, Poesen L, Zijlstra F, et al. To Uncertainty and Beyond: Identifying the Capabilities Needed by Hospitals to Function in Dynamic Environments. Medical Care Research and Review. 2022;79(4):549-61.

13. Alajmi A, Adlan N, Lahyani R. Assessment of Supply Chain Management Resilience within Saudi Medical Laboratories during Covid-19 Pandemic. Procedia Cirp. 2021;103:32-6.

14. Marshall F, Gordon A, Gladman JR, Bishop S. Care homes, their communities, and resilience in the face of the COVID-19 pandemic: interim findings from a qualitative study. BMC Geriatrics. 2021;21(1):1-10.

15. Spieske A, Gebhardt M, Kopyto M, Birkel H. Improving resilience of the healthcare supply chain in a pandemic: Evidence from Europe during the COVID-19 crisis. Journal of Purchasing and Supply Management. 2022:100748.

16. Lyng HB, Ree E, Wibe T, Wiig S. Healthcare leaders’ use of innovative solutions to ensure resilience in healthcare during the Covid-19 pandemic: a qualitative study in Norwegian nursing homes and home care services. BMC Health Services Research. 2021;21(1):1-11.

17. Ballantyne H, Achour N. The challenges of nurse redeployment and opportunities for leadership during COVID-19 pandemic. Disaster Medicine and Public Health Preparedness. 2022:1-7.

18. Akinyemi OO, Popoola OA, Fowotade A, Adekanmbi O, Cadmus EO, Adebayo A. Qualitative exploration of health system response to COVID-19 pandemic applying the WHO health systems framework: Case study of a Nigerian state. Scientific African. 2021;13:e00945.

19. Yoon S, Goh H, Chan A, Malhotra R, Visaria A, Matchar D, et al. Spillover effects of COVID-19 on essential chronic care and ways to foster health system resilience to support vulnerable non-COVID patients: a multistakeholder study. Journal of the American Medical Directors Association. 2022;23(1):7-14.

20. Danhieux K, Buffel V, Pairon A, Benkheil A, Remmen R, Wouters E, et al. The impact of COVID-19 on chronic care according to providers: a qualitative study among primary care practices in Belgium. BMC Family Practice. 2020;21(1):1-6.

21. Hodgins M, Van Leeuwen D, Braithwaite J, Hanefeld J, Wolfe I, Lau C, et al. The COVID-19 system shock framework: capturing health system innovation during the COVID-19 pandemic. International Journal of Health Policy and Management. 2022;11(10):2155-65.

22. Seruwagi G, Nakidde C, Otieno F, Kayiwa J, Luswata B, Lugada E, et al. Healthworker preparedness for COVID-19 management and implementation experiences: a mixed methods study in Uganda’s refugee-hosting districts. Conflict and Health. 2021;15(1):1-22.

23. Cannedy S, Bergman A, Medich M, Rose DE, Stockdale SE. Health system resiliency and the COVID-19 pandemic: a case study of a new nationwide contingency staffing program. Healthcare. 2022;10(2):244.

24. Nair S, Kannan P, Mehta K, Raju A, Mathew J, Ramachandran P. The COVID-19 pandemic and its impact on mental health services: the provider perspective. Journal of Public Health. 2021;43(Supplement_2):ii51-ii6.

25. Stengel S, Roth C, Breckner A, Cordes L, Weber S, Ullrich C, et al. Resilience of the primary health care system–German primary care practitioners’ perspectives during the early COVID-19 pandemic. BMC Primary Care. 2022;23(1):1-13.

26. Parikh N, Chaudhuri A, Syam SB, Singh P. Fostering Resilient Health Systems in India: Providing Care for PLHIV Under the Shadow of COVID-19. Frontiers in Public Health. 2022:1581.

27. Snowdon A, Wright A, editors. Digitally enabled supply chain as a strategic asset for the COVID-19 response in Alberta. Healthcare Management Forum; 2022: SAGE Publications Sage CA: Los Angeles, CA.

28. Juárez-Ramírez C, Reyes-Morales H, Gutiérrez-Alba G, Reartes-Peñafiel DL, Flores-Hernández S, Muños-Hernández JA, et al. Local health systems resilience in managing the COVID-19 pandemic: lessons from Mexico. Health Policy and Planning. 2022;37(10):1278-94.

29. Minka S-O, Minka F-H, Chauvin A, Revue E, Plaisance P, Casalino E, et al. Resilience strategy in emergency medicine during the Covid-19 pandemic in Paris. Journal Européen des Urgences et de Réanimation. 2021;33(2):88-95.

30. McCollum R, Zaizay Z, Dean L, Watson V, Frith L, Alhassan Y, et al. Qualitative study exploring lessons from Liberia and the UK for building a people-centred resilient health systems response to COVID-19. BMJ Open. 2022;12(8):e058626.

31. Saurin TA, Wachs P, Bueno WP, de Souza Kuchenbecker R, Boniatti MM, Zani CM, et al. Coping with complexity in the COVID pandemic: An exploratory study of intensive care units. Human Factors and Ergonomics in Manufacturing & Service Industries. 2022;32(3):301-18.
